# An Essential Role for (p)ppGpp in the Integration of Stress Tolerance, Peptide Signaling, and Competence Development in *Streptococcus mutans*

**DOI:** 10.3389/fmicb.2016.01162

**Published:** 2016-07-28

**Authors:** Justin Kaspar, Jeong N. Kim, Sang-Joon Ahn, Robert A. Burne

**Affiliations:** Department of Oral Biology, College of Dentistry, University of Florida, GainesvilleFL, USA

**Keywords:** competence, biofilms, *comX*, stringent response, (p)ppGpp, dental caries

## Abstract

The microbes that inhabit the human oral cavity are subjected to constant fluctuations in their environment. To overcome these challenges and gain a competitive advantage, oral streptococci employ numerous adaptive strategies, many of which appear to be intertwined with the development of genetic competence. Here, we demonstrate that the regulatory circuits that control development of competence in *Streptococcus mutans*, a primary etiological agent of human dental caries, are integrated with key stress tolerance pathways by the molecular alarmone (p)ppGpp. We first observed that the growth of a strain that does not produce (p)ppGpp (Δ*relAPQ*, (p)ppGpp^0^) is not sensitive to growth inhibition by *comX* inducing peptide (XIP), unlike the wild-type strain UA159, even though XIP-dependent activation of the alternative sigma factor *comX* by the ComRS pathway is not impaired in the (p)ppGpp^0^ strain. Overexpression of a (p)ppGpp synthase gene (*relP*) in the (p)ppGpp^0^ mutant restored growth inhibition by XIP. We also demonstrate that exposure to micromolar concentrations of XIP elicited changes in (p)ppGpp accumulation in UA159. Loss of the RelA/SpoT homolog (RSH) enzyme, RelA, lead to higher basal levels of (p)ppGpp accumulation, but to decreased sensitivity to XIP and to decreases in *comR* promoter activity and ComX protein levels. By introducing single amino acid substitutions into the RelA enzyme, the hydrolase activity of the enzyme was shown to be crucial for full *com* gene induction and transformation by XIP. Finally, loss of *relA* resulted in phenotypic changes to Δ*rcrR* mutants, highlighted by restoration of transformation and ComX protein production in the otherwise non-transformable Δ*rcrR*-NP mutant. Thus, RelA activity and its influence on (p)ppGpp pools appears to modulate competence signaling and development through RcrRPQ and the peptide effectors encoded within *rcrQ*. Collectively, this study provides new insights into the molecular mechanisms that integrate intercellular communication with the physiological status of the cells and the regulation of key virulence-related phenotypes in *S. mutans*.

## Introduction

The human oral cavity typically hosts over six billion microbes, with as many as 1,200 distinct taxa being identified in metagenomic studies (Jenkinson, 2011). Oral microbes contend with constant fluctuations in environmental conditions, including pH, oxygen tension, nutrient availability, and carbohydrate source (Lemos and Abranches, 2005). These dynamic perturbations can alter the composition and biochemical activities of the biofilms by disrupting a homeostatic environment and shifting the oral microbiome from a state of health to one of disease. Oral streptococci are particularly abundant members of the oral microbiota and have evolved numerous mechanisms to respond to these environmental challenges in a way that gives them a competitive advantage for persistence in oral biofilms (Lemos and Burne, 2008). Although many oral streptococci have been associated with oral health, *Streptococcus mutans* often shows the strongest association with human dental caries (Takahashi and Nyvad, 2011).

Under conditions of nutrient limitation, bacteria employ a regulatory mechanism known as the stringent response, which is characterized in part by inhibition of stable RNA, DNA, and cell wall synthesis, an increase in amino acid biosynthesis, and alterations in expression of stress genes (Carneiro et al., 2011). The stringent response is mediated by the nutritional alarmones guanosine 3′-diphosphate 5′-triphosphate and guanosine 3′,5′-bispyrophosphate, which together are referred to as (p)ppGpp (Dalebroux and Swanson, 2012). In the classical stringent response, originally characterized in the Gram-negative bacterium *Escherichia coli*, (p)ppGpp is synthesized by addition of pyrophosphate from ATP to either GTP or GDP by the RelA synthetase. RelA becomes active in response to deacylated tRNA molecules binding to the ribosomal A-site as amino acids pools are depleted, leading to abortion of translation when the ribosomes stall. In *E. coli*, the RelA enzyme has only synthetase activity, whereas the SpoT enzyme has weak synthetase activity and efficient (p)ppGpp hydrolase activity. In Gram-positive bacteria, synthetase and hydrolase activities are combined into single, bifunctional enzymes referred to as a Rel/SpoT homolog (RSH) or Rel enzymes (Atkinson et al., 2011). The dual catalytic domains of these bifunctional enzymes are located in the N-terminal domain of the proteins, while the C-terminal domain participates in allosteric regulation. Rel enzymes have the ability to switch between a synthetase-ON/hydrolase-OFF state and a synthetase-OFF/hydrolase-ON state (Mechold et al., 1996, 2002; Hogg et al., 2004). Deletion of the *relA* gene encoding the Rel enzyme of *S. mutans* was shown to alter biofilm formation and acid tolerance, and to affect the regulation of sugar catabolism (Lemos et al., 2004; Nascimento et al., 2008). In some bacteria, including *S. mutans*, there are additional (p)ppGpp synthetases, referred to as small alarmone synthetases (SAS), which lack (p)ppGpp hydrolase domains. The gene encoding the RelP synthetase of *S. mutans* is located in an operon with a two-component signal transduction system encoded by *relRS*. RelP is responsible for the majority of (p)ppGpp produced during exponential growth (Lemos et al., 2007). Another synthetase, RelQ, plays an important role in acid and oxidative stress tolerance. The *relQ* gene is co-transcribed with an NAD kinase (*ppnK*), a pseudouridine synthase (*rluE*), and a phosphotransacetylase (*pta*; Kim et al., 2012). Acetate metabolism and (p)ppGpp accumulation were recently shown to be tightly linked via the Pta-Acetate kinase pathway, and acetyl-phosphate accumulates under conditions of oxidative stress (Kim et al., 2015).

Seaton et al. (2011) characterized the *rcrRPQ* operon of *S. mutans*, encoding a MarR-like transcriptional repressor (RcrR) of the *rcrR* promoter and a pair of ABC exporters (RcrP, RcrQ), that exert significant control over (p)ppGpp production and the development of genetic competence. The gene cluster (SMU.921–923) is situated in close proximity to the *relPRS* operon (SMU.926–928), and certain mutations in *rcrRPQ* have negative effects on the expression of *relP* that lead to a reduction in (p)ppGpp pools during exponential growth (Seaton et al., 2011). Additionally, the expression of the *rcrRPQ* operon is significantly increased in response to accumulation of (p)ppGpp during a mupirocin-induced stringent response (Nascimento et al., 2008). Thus, regulation of *rcrRPQ* and (p)ppGpp accumulation are well integrated in *S. mutans*. In terms of genetic competence, the impact of deletion-replacement mutations of *rcrR* is strongly dependent on the polarity of the inserted marker, which greatly influences the expression levels of the ABC transporter genes, as well as of two peptide effectors encoded in the 3′ region of *rcrQ* (Ahn et al., 2014; Seaton et al., 2015). A polar mutation within *rcrR* (Δ*rcrR*-P) is constitutively hyper-transformable, but a non-polar mutation (Δ*rcrR*-NP) is non-transformable. The non-transformable state of the Δ*rcrR*-NP strain is correlated with overexpression of the *rcrPQ* gene and *rcrQ* peptides, and is characterized by a dramatic decrease in the amount of mRNA detectable from the 5′ portion of *comX* (or σ^X^), which encodes the alternative sigma factor that is the master regulator of late competence gene expression that is absolutely required for cells to progress to competence. Interestingly, the loss of full-length *comX* mRNA and ComX protein in the Δ*rcrR*-NP mutant is accompanied by the activation of expression of an open reading frame (ORF) internal to *comX* that codes for XrpA (ComX regulatory peptide A; Kaspar et al., 2015). If point mutations that block the production of XrpA are present in the Δ*rcrR*-NP genetic background, expression of a full-length *comX* transcript, ComX protein levels and transformability are all restored to levels similar to the Δ*rcrR*-P strain (Kaspar et al., 2015). Although genetic studies clearly show that RcrRPQ, XrpA and the *rcrQ*-associated peptide effectors work in concert to control competence development, strongly influencing *comX* expression and ComX protein levels, the mechanisms by which these effectors exert their influence on the competence cascade has not yet been defined.

The early stages of competence development in *S. mutans* require a complex signaling network that ultimately leads to activation of expression of the gene for ComX. ComX directs transcription of a regulon of late competence genes, many of which are responsible for the uptake of exogenous DNA from the environment and homologous recombination into the bacterial chromosome, when possible (Cvitkovitch, 2001; Merritt et al., 2005b). Regulation of *comX* expression is modulated directly by the ComRS system (Fontaine et al., 2009; Mashburn-Warren et al., 2010). The *comS* gene encodes a 17-aa peptide that is exported and processed by an unknown mechanism into the active, 7-aa *comX* inducing peptide (XIP); XIP is composed of the C-terminal 7 residues of ComS. Externalized XIP is imported by the oligopeptide permease OppA, an ABC transporter, and is bound by the Rgg-like transcriptional activator ComR (Desai et al., 2012; Khan et al., 2012). The ComR–XIP complex activates the promoters for *comX* and *comS*, the latter creating a positive feedback loop (Fontaine et al., 2013). Provision of relatively high concentrations of XIP (2–10 μM) to *S. mutans* can also induce cell death and result in apparent slow growth of the organism (Wenderska et al., 2012). However, the molecular basis for XIP-mediated cell death or growth inhibition is not currently understood. Activation of *comX* expression by XIP and the ComRS system in a chemically defined medium shows a unimodal response across the entire population (Son et al., 2012), and can be influenced by environmental pH (Guo et al., 2014; Son et al., 2015a). The ComCDE pathway of *S. mutans* also induces *comX* expression in a bimodal fashion in complex media that contains peptides, albeit via an indirect mechanism (Hung et al., 2011; Reck et al., 2015; Son et al., 2015b).

In addition to the ComCDE and ComRS pathways, a variety of other gene products in *S. mutans* can impact transformation efficiency by altering early and/or late competence gene expression, including VicRK, HtrA, CiaRH, HdrM, and others (Ahn et al., 2005; Merritt et al., 2005a; Senadheera et al., 2005, 2012; Abranches et al., 2006; Niu et al., 2008; Banu et al., 2010; Okinaga et al., 2010; Kajfasz et al., 2011; Mair et al., 2012; Kim et al., 2013). Many of these regulatory systems that affect competence have been shown to be critical for adaptation to environmental stress, so there is a considerable body of evidence demonstrating that induction of competence and stress tolerance are tightly intertwined. It was previously reported that there was no reduction in transformation efficiency associated with deletion of *relA* in *S. mutans* when cells were made competent in complex medium in the presence or absence of competence stimulating peptide (CSP) (Lemos et al., 2004). However, the recent evidence showing that *rcrRPQ* gene products affect both (p)ppGpp metabolism and genetic competence, coupled with the discovery of the *comRS* pathway and its direct and essential role in activation of *comX* expression call for a reevaluation of the potential linkage of (p)ppGpp metabolism with competence and its relationship to the RcrRPQ system. We report here that gene products involved in (p)ppGpp metabolism can dominantly impact the development of genetic competence and reverse the effect of an *rcrR* mutation that eliminates transformability, providing critical new insights into the regulation of competence and virulence traits by *S. mutans*.

## Materials and Methods

### Bacterial Strains and Growth Conditions

*Escherichia coli* strain DH10B was grown in Luria broth that was supplemented with 300 μg ml^-1^ erythromycin and 50 μg ml^-1^ of kanamycin or spectinomycin, when needed. *S. mutans* wild-type (WT) strain UA159 and its derivatives (**Table 1**) were grown in either brain heart infusion (BHI; Difco) or FMC medium (Terleckyj and Shockman, 1975) that was supplemented with 10 μg ml^-1^ erythromycin and 1 mg ml^-1^ of kanamycin or spectinomycin, as needed. Synthetic XIP (sXIP, aa sequence = GLDWWSL), corresponding to residues 11–17 of ComS, was synthesized and purified to 96% homogeneity by NeoBioSci (Cambridge, MA, USA). The lyophilized sXIP was reconstituted with 99.7% dimethyl sulfoxide (DMSO) to a final concentration of 2 mM and stored in 40 μl aliquots at -20°C. Unless otherwise noted, cultures were grown overnight in BHI medium with antibiotics, if needed, at 37°C in a 5% CO_2_ aerobic atmosphere. The cultures were harvested by centrifugation, washed in 1 mL FMC, and resuspended in FMC to remove all traces of BHI before fresh medium was inoculated. For growth rate comparisons, overnight cultures were diluted 1:50 and grown to mid-exponential phase (OD_600_ = 0.5–0.6). Then, cultures were re-diluted 1:100 into 300 μl of FMC and added to multiwell plates, sXIP was added to the desired final concentrations (0.2, 2, or 20 μM), and cultures were overlaid with 50 μl of sterile mineral oil to reduce the growth inhibitory effects of air on growth of *S. mutans*. The optical density at 600 nm (OD_600_) was monitored at 30 min intervals for 24 h using a Bioscreen C lab system (Helsinki, Finland) at 37°C with shaking for 10 s before each reading.

**Table 1:** List of strains.

Strains and plasmids used in this study		
**Strain or plasmid**	**Relevant characteristic(s)^∗^**	**Source or reference**

**Strains**		
UA159	Wild-type	ATCC 700610
(p)ppGpp^0^ (Δ*relAPQ*)	Δ*relA*Δ*relP*Δ*relQ*::Erm^R^ Sp^R^ NPKm^R^	Lemos et al. (2007)
184 (p)ppGpp^0^	Δ*relP*Δ*relQ*::Sp^R^ NPKm^R^, 264th aa *relA* D:G harboring pIB184	This study
184RelP (p)ppGpp^0^	Δ*relP*Δ*relQ*::Sp^R^ NPKm^R^, 264th aa *relA* D:G harboring pIB184	This study
Δ*relA*	Δ*relA*::ΩErm^R^	Lemos et al. (2004)
Δ*relPQ*	Δ*relP* Δ*relQ*::Sp^R^ NPKm^R^	Lemos et al. (2007)
Δ*relP*	Δ*relP*::NPKm^R^	Lemos et al. (2007)
Δ*relQ*	Δ*relQ*::NPKm^R^	Lemos et al. (2007)
P*comR*-LacZ	UA159::P*comR*-LacZ, Km^R^	This study
P*comR*-LacZ/Δ*relA*	Δ*relA*::P*comR*-LacZ, Km^R^Erm^R^	This study
P*comX*-LacZ	UA159::P*comX*-LacZ, Km^R^	Son et al. (2012)
P*comX*-LacZ/Δ*relA*	Δ*relA*::P*comX*-LacZ, Km^R^Erm^R^	This study
*relA*+/Δ*relA*	Δ*relA*::ΩKm^R^ harboring pMSP3535-*relA*	Lemos et al. (2004)
*relP*+/Δ*relA*	Δ*relA*::ΩKm^R^ harboring pMSP3535-*relP*	This study
184ComS/Δ*relA*	Δ*relA*::ΩErm^R^ harboring pIB184-*comS*	This study
184ComR/Δ*relA*	Δ*relA*::ΩErm^R^ harboring pIB184-*comR*	This study
184ComX/Δ*relA*	Δ*relA*::ΩErm^R^ harboring pIB184-*comX*	This study
*relA*^Δ^*^SY N^*	264th aa *relA* D:G	This study
*relA*^Δ^*^SY N^*^ΔHY D^	151st aa *relA* T:P; 264th aa *relA* D:G	This study
*relA^385^*	Stop codon (TAA) insertion at 385th aa	This study
Δ*rcrR*-P	*rcrR*::ΩKm^R^	Seaton et al. (2011)
Δ*rcrR*-PΔ*relA*	rcrR::ΩKm^R;^ Δ*relA*::ΩErm^R^	This study
Δ*rcrR*-NP	*rcrR*::NPKm^R^	Seaton et al. (2011)
Δ*rcrR*-NPΔ*rel A*	rcrR::NPKm^R;^ Δ*relA*::ΩErm^R^	This study
**Plasmids**		
pDL278	*E. coli–Streptococcus* shuttle vector, Sp^R^	LeBlanc et al. (1992)
pMSP3535	*E. coli–Streptococcus* shuttle vector, Erm^R^	Bryan et al. (2000)
pIB184	*Streptococcus* shuttle vector, Erm^R^	Biswas et al. (2008)

*^∗^Ω, polar; NP, non-polar; Km, kanamycin; Erm, erythromycin; Sp, spectinomycin.*

### Construction of Strains and DNA Manipulation

Mutant strains of *S. mutans* were created using a PCR ligation mutagenesis approach (Supplementary Table S1) (Lau et al., 2002). Splice overlap extension was utilized to create single base change mutations within the *relA* gene using protocols described elsewhere (Ho et al., 1989; Cha et al., 1992; Kaspar et al., 2015). Overexpression of genes was achieved by amplifying the genes of interest from *S. mutans* UA159 and cloning into the expression vector pIB184 (Biswas et al., 2008; Guo et al., 2014; Kaspar et al., 2015). Transformants were confirmed by PCR and sequencing after selection on BHI agar containing the appropriate antibiotic(s). Plasmid DNA was isolated from *E. coli* by using QIAGEN (Chatsworth, CA, USA) columns, and restriction and DNA-modifying enzymes were obtained from Invitrogen (Gaithersburg, MD, USA) or New England Biolabs (Beverly, MA, USA). PCRs were carried out with 100 ng of chromosomal DNA using Taq DNA polymerase, and PCR products were purified with the QIAquick kit (QIAGEN). DNA was introduced into *S. mutans* by natural transformation and into *E. coli* by the calcium chloride method (Cosloy and Oishi, 1973).

### RNA Extraction and Quantitative Real-Time PCR

To measure the expression of genes using quantitative real-time PCR (RT-qPCR), three colonies of *S. mutans* WT (UA159) and the mutant strains of interest (Δ*relAPQ*, Δ*relA*) were grown overnight and a 1:50 dilution was added to fresh FMC medium the next day. When cells reached an OD_600_ of 0.2, cultures were adjusted to a final concentration of 1% DMSO or 2 μM sXIP and incubated at 37°C in a 5% CO_2_ atmosphere for 1 h before being harvested by centrifugation. Cell lysis was achieved through mechanical disruption (bead beating) and RNA was extracted by acidic phenol phase separation. The RNA was further purified with an RNeasy mini kit (QIAGEN) according to the provided protocol, and treatment with DNase 1 (QIAGEN). Purified RNA (1 μg) was used to generate cDNA from gene-specific primers (Supplementary Table S1) using the Superscript III first-strand synthesis (Invitrogen) reverse transcription protocol. Real-time PCRs were carried out using an iCyclerQ real-time PCR detection system (Bio-Rad) and iQ SYBR green supermix (Bio-Rad) according to the supplier’s protocol. 16S rRNA was used as an internal reference. All assays were performed in triplicate with RNA isolated from three biological replicates.

### Transformation Assays

Strains of *S. mutans* were grown overnight and then diluted 1:50 in 200 μl of FMC medium. When the cultures reached an OD_600_ = 0.2, either a final concentration of 1% DMSO or 2 μM sXIP was added to the growing culture. After 10 min of incubation, 500 ng of purified plasmid pDL278, which harbors a spectinomycin resistance (Sp^R^) gene, was added. Following 3 h of further incubation, dilutions of the cultures were plated on BHI agar plates with or without 1 mg ml^-1^ spectinomycin. Colony forming units (CFUs) were counted after 48 h. Transformation efficiency was determined by dividing the number of Sp^R^ transformants by the total number of CFU recovered on non-selective medium.

### Western Blotting

Overnight cultures of *S. mutans* were diluted 1:50 into 35 mL of fresh FMC medium and harvested by centrifugation when the cultures reached an OD_600_ = 0.5. When desired, 1% DMSO or 2 μM sXIP (final concentrations) was added when the cultures reached an OD_600_ value of 0.2. Cell pellets were washed once with buffer A (0.5 M sucrose; 10 mM Tris–HCl, pH 6.8; 10 mM MgSO_4_) containing 10 μg ml^-1^ of phenylmethanesulfonyl fluoride (PMSF; ICN Biomedicals Inc.), collected by centrifugation, and resuspended in 0.5 mL Tris-buffered saline (50 mM Tris–HCl, pH 7.5; 150 mM NaCl). Cells were lysed using a Mini Bead Beater (Biospec Products) in the presence of 1 volume of glass beads (average diameter 0.1 mm) for 30 s intervals, three times, with incubation on ice between homogenizations. Lysates were then centrifuged at 3,000 ×*g* for 10 min at 4°C. Protein concentrations of the resulting supernates were determined using the bicinchoninic acid assay (BCA; Thermo Scientific) with purified bovine serum albumin as the standard. ComX protein was detected by immunoblotting using a protocol detailed elsewhere (Kaspar et al., 2015). Protein preparations (10 μg) were loaded on a 15% polyacrylamide gel with a 6% stacking gel and separated by sodium dodecyl sulfate-polyacrylamide gel electrophoresis (SDS-PAGE), transferred to Immobilon-P polyvinylidene difluoride membranes (Millipore), then treated with primary polyclonal anti-ComX antisera at a 1:1,000 dilution and a secondary peroxidase-labeled, goat anti-rabbit IgG antibody (1:5,000 dilution; Kirkegaard & Perry Laboratories, USA). Detection was performed using a SuperSignal West Pico Chemiluminescent Substrate kit (Thermo Scientific) and visualized with a FluorChem 8900 imaging system (Alpha Innotech, USA).

### β-Galactosidase Assays

For measurements of LacZ produced from promoter:reporter gene fusions, strains were grown overnight, washed in FMC, resuspended, and diluted 1:40 into fresh FMC medium. At OD_600_ = 0.2, sXIP was added to a final concentration of 2 μM, cells were incubated for an additional 90 min, then cells were harvested by centrifugation. β-galactosidase activity was measured by using a modification of the Miller protocol (Zubay et al., 1972). Briefly, cells were washed once with Z buffer [Na-phosphate buffer (pH 7.0), 10 mM KCl, 1 mM MgSO_4_, 5 mM β-mercaptoethanol), and resuspended in 1.3 ml Z buffer. Part of the sample (500 μl) was vortexed with 25 μl of a toluene–acetone mix (1:9) for 2 min and kept at 37°C, the remaining 0.8 ml of the suspension was used to determine OD_600_. The LacZ reaction was initiated by the addition of 100 μl of *o*-nitrophenyl-β-D-galactopyranoside (4 mg ml^-1^) and was terminated by the addition of 500 μl of 1 M Na_2_CO_3_. Samples were centrifuged for 1 min using a tabletop centrifuge and the OD of the supernatant fluid was measured at 420 and 550 nm. Activity was expressed in Miller units (Zubay et al., 1972).

### Monitoring (p)ppGpp Accumulation

Visualization of (p)ppGpp accumulation was conducted as described elsewhere (Lemos et al., 2007). Briefly, overnight cultures from strains of interest were diluted 1:50 into a modified FMC medium containing a lower concentration of inorganic phosphate (Lemos et al., 2007). When cells reached an OD_600_ of 0.2, [^32^P]-orthophosphate, along with the desired amount of sXIP, was added. Cells were then incubated at 37°C for the specified duration, then harvested by centrifugation. (p)ppGpp was extracted by addition of an equal volume of ice-cold 13 M formic acid, followed by two freeze–thaw cycles in a dry ice/ethanol bath. After extraction, the CPM μl^-1^ of the supernatant fraction was measured in a scintillation counter and 2.0 × 10^5^ CPM of each sample was spotted onto a polyethyleneimine (PEI) cellulose plate (Selecto Scientific) for thin-layer chromatography (TLC). The TLC plates were chromatographed in one dimension with 1.5 M KH_2_PO_4_ (adjusted to pH 3.4 with phosphoric acid), air-dried and exposed to X-ray film at -70°C. The signal density of spots corresponding to ppGpp (GP4) and pppGpp (GP5) was analyzed using ImageJ (v1.47) software^1^.

## Results

### A (p)ppGpp^0^ Mutant of *S. mutans* Displays Minimal Growth Inhibition by XIP

**FIGURE 1:**
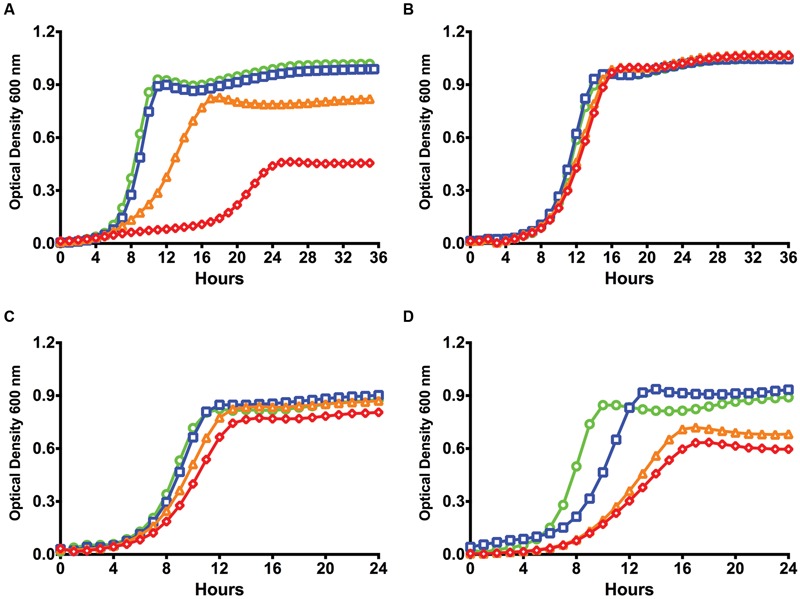
**Lack of (p)ppGpp abrogates growth inhibition by XIP.** Growth curves of **(A)** *S. mutans* UA159, **(B)** a (p)ppGpp^0^ derivative of UA159 (Δ*relAPQ*), **(C)** pIB184 vector only in the (p)ppGpp^0^ genetic background, and **(D)** pIB184RelP in the (p)ppGpp^0^ genetic background in the presence of different concentrations of sXIP. Bacterial cultures were grown in the chemically defined medium FMC containing 1% DMSO (●; green), or sXIP at final concentrations of 0.2 μM (■; blue), 2 μM (▲; orange), or 20 μM (◆; red). sXIP was dissolved in DMSO, so the final concentration of DMSO in all samples was 1%.

While previous communications indicated that a deletion of the *relA* gene of *S. mutans* does not appear to have effects on transformation efficiency (Lemos et al., 2004), those experiments were performed in the complex medium BHI in the presence of CSP. Since that time, it has come to light that the ComRS system is the proximal regulator of alternative sigma factor *comX* expression, not ComDE, in *S. mutans*. Importantly, the ComRS system has been shown to function optimally in a chemically defined medium (Son et al., 2012), and requires ComS-derived XIP for ComR-dependent activation of gene expression (Mashburn-Warren et al., 2010). To determine if (p)ppGpp influenced the development of competence by ComRS in a chemically defined medium (FMC), we compared the growth kinetics between the *S. mutans* WT strain UA159 and a (p)ppGpp^0^ mutant (Δ*relAPQ*) in the presence of various concentrations of sXIP, which was dissolved in DMSO. Cells treated with the same final v/v concentration (1%) of DMSO served as controls. The WT strain UA159 displayed apparent growth inhibition, as assessed by monitoring doubling time and final optical density, as the concentration of sXIP was increased (Wenderska et al., 2012; Guo et al., 2014; **Figure 1A**). We specify apparent growth inhibition because cell lysis may also influence the total optical density at any given time point. The exponential doubling time of UA159 increased from 66 ± 1 to 204 ± 5 min as the concentration of sXIP was increased from 0.2 to 2 μM. A doubling time of 229 ± 18 min was observed in the presence of 20 μM sXIP. A reduction in the final yield (final OD_600_ at the 36-h time point) was seen with increasing concentrations of sXIP; 1.08 ± 0.01 with no XIP and 0.45 ± 0.08 at the highest concentration of XIP. Interestingly, the growth inhibitory effects of sXIP were not observed in the (p)ppGpp^0^ strain (**Figure 1B**). As sXIP concentrations were increased from 0.2 μM to 2 or 20 μM, there were only minimal increases in the doubling times: 98 ± 2, 111 ± 1, and 109 ± 2 min, respectively. Similarly, there were only marginal reductions in the final yields in the strain lacking (p)ppGpp.

To confirm that changes in growth inhibition were due to a loss of (p)ppGpp, we added back the *relP* gene, encoding a SAS that lacks hydrolase activity, under the control of the constitutive P23 promoter on a shuttle plasmid (Biswas et al., 2008) and compared the strains growth kinetics to one containing only the empty pIB184 vector. While the (p)ppGpp^0^ strain containing the empty vector showed limited growth inhibition in the presence of XIP (**Figure 1C**), addition of *relP* led to increased doubling times of 208 ± 13 and 273 ± 24 min with addition of 2 or 20 μM sXIP, respectively, compared to 86 ± 3 min with the 1% DMSO control (**Figure 1D**). Additionally, final yields dropped from 0.88 ± 0.02 at 24 h after growth without sXIP to 0.68 ± 0.03 and 0.60 ± 0.03 with 2 or 20 μM sXIP. These data suggest that modulation of (p)ppGpp levels, as seen with the complementation of the (p)ppGpp^0^ strain, can lead to phenotypic changes in response to the competence inducing peptide XIP, altering the response level to the signaling peptide.

This lack of growth inhibition phenotype in cells treated with sXIP is commonly associated with strains that have mutations in either *comR* or *comX*, indicating a severe reduction in early *com* gene expression that would lead to the (p)ppGpp^0^ failing to respond to the sXIP signal (Perry et al., 2009; Wenderska et al., 2012). This same phenotype is also observed in the Δ*rcrR*-NP strain, which fails to express a full-length *comX* mRNA (Kaspar et al., 2015). To test for *com* gene down-regulation, expression was measured by qRT-PCR in the WT and (p)ppGpp^0^ strains 1 h after addition of 2 μM sXIP to cells at OD_600__nm_ = 0.2. Interestingly the *comX* gene, encoding the alternative sigma factor required for late competence gene expression (Supplementary Figure S1A), and the late competence gene *comYA* (Supplementary Figure S1B) were both induced to the same level in the mutant and WT strains treated with XIP. The qRT-PCR results were consistent with the finding that similar levels of ComX protein (Kaspar et al., 2015) were present in lysates of mutant and WT cells that had been treated with XIP (Supplementary Figure S1C). Similar gene expression profiles between the WT and (p)ppGpp^0^ strains following addition of sXIP were also observed for a direct target of ComE (*comD*), for the *comRS* genes, and for *rcrR*, the first gene in the *rcrRPQ* operon that has been shown to influence competence, stress tolerance, and (p)ppGpp accumulation (Supplementary Figures S1D–G). Thus, the ComRS signaling pathway was intact and functional in both the WT and (p)ppGpp^0^ strains, so the specific impact of complete loss of (p)ppGpp on the growth and competence phenotypes apparently does not occur at the level of *comRS* expression or formation of an active ComR–XIP complex.

### (p)ppGpp Levels Are Influenced by XIP Exposure

**FIGURE 2:**
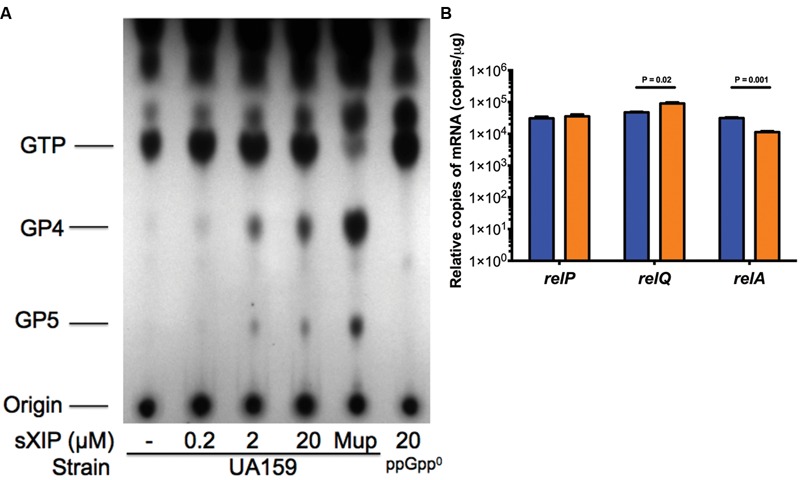
**Accumulation of (p)ppGpp with addition of sXIP. (A)** (p)ppGpp accumulation in *S. mutans* UA159 with various concentrations of sXIP. Cells were labeled with ^32^P-orthophosphate in FMC medium when OD_600__nm_ reached 0.15, along with addition of either 1% DMSO, 0.2, 2, or 20 μM sXIP. After an hour, cells were harvested. Nucleotides were extracted by addition of 13 M formic acid, followed by three freeze–thaw cycles. Resulting supernates were spotted onto PEI-cellulose plates for TLC in 1.5 M KH_2_PO_4_. Identity of the migrating nucleotides is shown to the left. Mupirocin (Mup; 50 μg ml^-1^), previously shown to induce a potent stringent response, serves as the positive control for (p)ppGpp accumulation. **(B)** Measurement of *relP, relQ*, and *relA* expression by qRT-PCR of UA159 with addition of either 1% DMSO (blue bars) or 2 μM sXIP (orange bars).

Clearly, (p)ppGpp levels can alter the growth phenotype of cells exposed to sXIP, so we reasoned that addition of sXIP may lead to an increase in internal (p)ppGpp pools. To determine how (p)ppGpp accumulation was affected by the addition of exogenous XIP, the WT strain UA159 was grown in FMC medium and labeled with ^32^P-orthophosphate in the presence of either 1% DMSO (control) or increasing concentrations of sXIP. More specifically, cultures of *S. mutans* were grown to OD_600_ = 0.15 and then ^32^P-orthophosphate and sXIP were added together. Cultures were incubated for 60 min, cells were harvested, and formic acid extracts of the phosphorylated guanosine nucleotides were prepared and separated by thin layer chromatography (**Figure 2A**). Addition of mupirocin, which leads to RelA-dependent accumulation of (p)ppGpp, served as a positive control. Only a very modest increase in (p)ppGpp accumulation was evident with addition of 0.2 μM sXIP, compared to the 1% DMSO control. However, there were substantial increases in (p)ppGpp pools in the samples treated with 2 or 20 μM sXIP (**Table 2**). The latter concentrations of sXIP correspond to those that induce the greatest degree of growth inhibition in UA159, providing further evidence of an essential role for (p)ppGpp in XIP-dependent inhibition of growth. Induction of (p)ppGpp after sXIP exposure is also time-dependent, as cells harvested at 10, 20, 40, and 60 min after addition of ^32^P-orthophosphate and sXIP showed the greatest accumulation of (p)ppGpp at the 40 and 60 min time points (Supplementary Figure S2). To determine how addition of sXIP to the growth medium might influence expression of individual *rel* genes, we measured the expression of *relP, relQ*, and *relA* mRNA levels in UA159 after addition of 2 μM sXIP (**Figure 2B**). Expression of the *relQ* gene was increased 1.9-fold after sXIP addition, compared to the 1% DMSO control, whereas *relA* gene expression was decreased 2.8-fold with XIP treatment. There were no increases noted in *relP* mRNA associated with sXIP treatment. However, it was recently reported that there is a ComE binding site in front of the *cipI* gene (SMU.925, *immB*; Khan et al., 2016), so we cannot rule out that ComE-dependent activation of *cipI* following XIP treatment might influence downstream *relP* (SMU.926) mRNA levels under other conditions that lead to activation of competence. Collectively, though, the results indicate increased production of RelQ and possible allosteric modulation of RelA synthetase/hydrolase activity can impact the quantity of (p)ppGpp in XIP-treated cells.

**Table 2:** Summary of (p)ppGpp-spot density detected in this study.

Samples	(p)ppGpp-spot density^a^	
	ppGpp	pppGpp
DMSO^b^	16.7 ± 4.5ˆc	NA^d^
0.2 μmol sXIP	29.3 ± 3.2	15.3 ± 2.5
2 μmol sXIP	117.7 ± 17.6, P < 0.01ˆe	60.0 ± 8.2
20 μmol sXIP	152.3 ± 8.0, P < 0.01	69.0 ± 11.3
50 μg.ml^-1^ mupirocin	215.0 ± 1.0, P < 0.01	145.3 ± 17.6
(p)ppGpp^0^ + 20 μmol sXIP	NA	NA

*^a^Sum of pixel in area. ^b^1% DMSO control, no addition of XIP. ^c^A numerical value that shows the means ± standard deviations of individual spot density. ^d^NA, not available. ^e^A statistical value, differs from the value of NO (Student’s *t*-test).*

### The RelA Enzyme Is Required for Optimal sXIP Signaling

**FIGURE 3:**
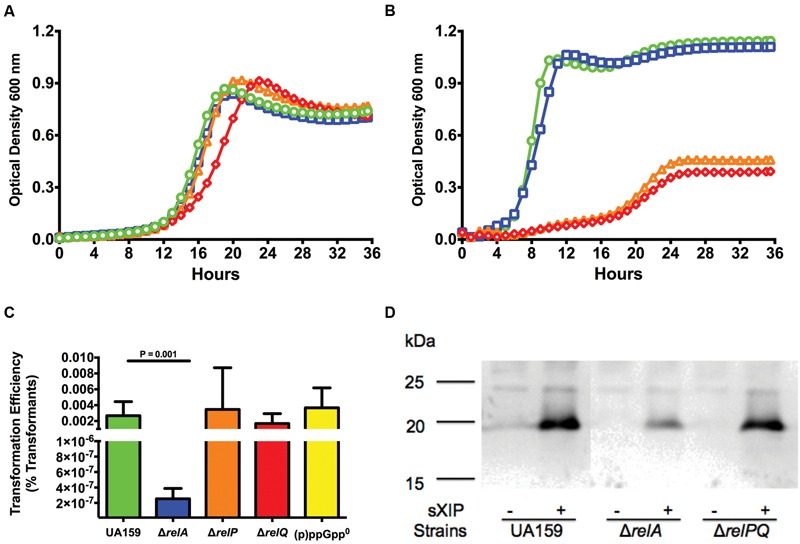
**Loss of *relA* results in decreases in sensitivity to XIP and transformation efficiency.** Growth curves of **(A)** Δ*relA* and **(B)** Δ*relPQ* strains treated with different concentrations of sXIP. Bacterial cultures were grown in the chemically defined medium FMC containing 1% DMSO (●; green), or 0.2 μM (■; blue), 2 μM (▲; orange), or 20 μM (◆; red) sXIP as in **Figure 1**. **(C)** Transformation efficiency of *S. mutans* UA159 (green bar), Δ*relA* (blue bar), Δ*relP* (orange bar), Δ*relQ* (red bar), and (p)ppGpp^0^ (Δ*relAPQ*; yellow bar) in FMC with addition of 2 μM sXIP. sXIP and plasmid pDL278 (Sp^R^) were added when OD_600_ reached 0.2. Serial dilutions of the cultures were plated on BHI agar with and without spectinomycin. Transformation efficiency was determined by the CFU of transformants divided by the total CFU of viable bacteria, multiplied by 100 (percent transformants). Data are the averages of three individual experiments with three biological replicates. Statistical analysis was performed by Student’s *t*-test. **(D)** Detection of ComX in UA159, Δ*relA*, and Δ*relPQ* lysates from cells grown in FMC with either 1% DMSO (-) or 2 μM sXIP (+), added when OD_600__nm_ reached 0.2 and followed by 1 h of incubation prior to harvesting and preparation of cell lysates. ComX was detected using a 1:5000 dilution of primary antiserum raised against full-length recombinant ComX from *S. mutans*. Molecular mass standards (in kDa) are shown to the left. The calculated molecular mass of ComX is 19 kDa.

The three (p)ppGpp synthetases in *S. mutans* appear to contribute to accumulation of alarmone under different circumstances (Lemos et al., 2007), so we explored the behavior of strains carrying individual or selected combinations of RSH and SAS enzymes on competence pathway signaling through ComRS. First, the growth kinetics of strains harboring individual deletion:replacement mutations of *relA* (**Figure 3A**), *relP*, and *relQ* were evaluated. The Δ*relA* strain showed the lowest sensitivity to sXIP, with a doubling time of 99 ± 5 to 101 ± 4 min in the presence of 0.2 and 2 μM sXIP, respectively. A longer doubling time (147 ± 3 min) was observed with 20 μM sXIP. Although doubling times increased with increased concentrations of sXIP, the final yields did not differ (0.70 ± 0.06 to 0.77 ± 0.03 min). In contrast, a strain carrying mutations in both *relP* and *relQ* (Δ*relPQ*) showed growth inhibitory effects to XIP that were similar to those seen with the WT strain (**Figure 3B**), as was the case for the strains carrying only *relP* or *relQ* mutations (data not shown). The observed doubling times for the Δ*relPQ* mutant strain were substantially greater than for the parental strain in the presence of 2 μM (229 ± 39 min) or 20 μM XIP (324 ± 68 min) and final yields were also lower (0.47 ± 0.14, 0.39 ± 0.05). Of note, a lag time of approximately 18 h was seen with 2 μM XIP in the Δ*relPQ* strain. When mutants lacking the *relA* gene were combined with individual *relP* or *relQ* mutations (Δ*relAP*, Δ*relAQ*), the strains grew like the Δ*relA* single mutant, providing further evidence that the loss of the Rel (RSH) enzyme alone could alter sensitivity to sXIP.

Fluctuations in sensitivity of strains to sXIP can be a potential indicator for changes in the ability to be transformed by exogenous DNA (Ahn et al., 2014; Kaspar et al., 2015). Transformation efficiency was measured in the Δ*relA*, Δ*relP*, and Δ*relQ* strains grown in FMC and treated with 2 μM XIP to induce competence. The Δ*relA* mutant strain showed a five-log reduction in transformation efficiency (2.0 × 10^-7^), compared to the WT strain (3.8 × 10^-2^; **Figure 3C**). In contrast to the Δ*relA* mutant, the Δ*relP* and Δ*relQ* strains showed no reduction in transformation efficiency. To confirm the results of the transformation assay, ComX levels were measured by western blot in the Δ*relA* and Δ*relPQ* strains. A clear decrease in ComX protein was seen in the Δ*relA* strain when treated with sXIP, but ComX protein in the Δ*relPQ* and WT strains were similar (**Figure 3D**). Interestingly, the Δ*relA* strain showed a decrease in transformation efficiency of less than one log compared to the WT strain when cells were grown in BHI medium and synthetic competence stimulating peptide (sCSP) was used to induce competence (3.7 × 10^-3^ to 1.2 × 10^-2^), comparable to the behavior noted for the Δ*relP* strain (3.4 × 10^-3^; Supplementary Figure S3). It is of interest that the effects of loss of the RSH enzyme were more profound in chemically defined medium. In particular, XIP is known to be ineffective at inducing *com* gene expression or enhancing transformation in complex medium (BHI), whereas sCSP only induces *comX* and competence in complex medium. Thus, it is likely that the effects of loss of the RSH enzyme are exerted through the ComRS pathway in a WT genetic background. Importantly, the behavior of the WT strain contrast dramatically with that of the (p)ppGpp^0^ strain, in which *com* gene expression was not similarly impacted. This difference in behavior is likely attributable to the fact that the (p)ppGpp^0^ strain completely lacks (p)ppGpp, whereas the WT and *relA* mutant have higher basal levels of (p)ppGpp due to a lack of (p)ppGpp hydrolase activity. Thus, *com* gene expression may only be influenced once (p)ppGpp production reaches a critical threshold and is not impacted when only low levels of (p)ppGpp are present, as is the case for the (p)ppGpp^0^ strain; highlighting further the critical role of (p)ppGpp in manifestation of these important phenotypes.

### *comR* Expression is Affected by the Loss of RelA

**FIGURE 4:**
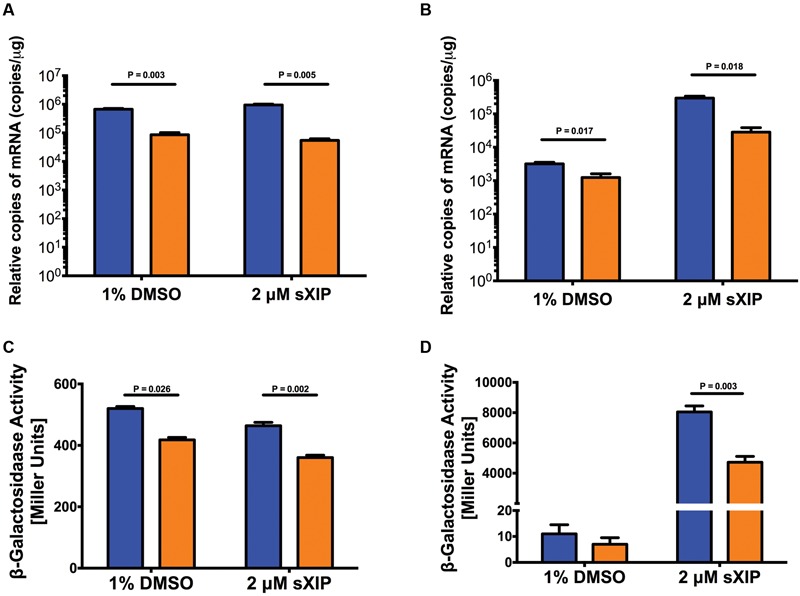
**Impacts of loss of *relA* on *com* gene expression.** Expression of *comR* **(A)** and *comX* **(B)** in *S. mutans* UA159 (blue bars) and its Δ*relA* mutant derivative (orange bars) after addition of 2 μM sXIP. sXIP was added when OD_600__nm_ reached 0.2. After 1 h of incubation, cells were harvested by centrifugation, RNA isolated, and qRT-PCR performed. Gene expression was normalized to 16S rRNA expression. Data represents three biological replicates measured in triplicate. Statistical analysis was performed by Student’s *t*-test. **(C,D)** Promoter activity from *lacZ* gene fusions to P*comR* **(C)** or P*comX* **(D)** in the wild-type (blue bars) and Δ*relA* (orange bars) genetic backgrounds determined by β-galactosidase (LacZ) activity in cells grown in FMC and treated with either 1% DMSO or 2 μM sXIP. sXIP was added when OD_600__nm_ reached 0.2. After an hour of incubation, cells were harvested by centrifugation and LacZ assays performed. Data represent three biological replicates assayed in triplicate. Statistical analysis was performed by Student’s *t*-test.

To determine where in the competence signaling cascade the effects of deletion of *relA* were occurring, the expression of selected *com* genes was measured by RT-qPCR in cells growing in FMC and treated with 2 μM sXIP. Expression levels of *comR* were one-log higher in UA159 than in the strain lacking the RSH enzyme (Δ*relA*), both in control (1% DMSO) samples and in cells treated with sXIP (**Figure 4A**). The levels of *comR* expression were not altered by exposure to XIP in either strain. A different pattern of expression was noted for *comX* (**Figure 4B**), *comYA* (Supplementary Figure S4A) and *comS* (Supplementary Figure S4B). In particular, expression of *comX* in the *relA*-deficient strain was modestly lower than in WT cells in the absence of sXIP treatment and about 1-log lower after treatment with XIP; a similar pattern was noted for *comYA*. It is significant, though, that induction of *comX* and *comYA* still occurs in response to sXIP treatment in the *relA*-deficient strain, albeit less so than in the WT genetic background. In contrast, the expression of *comS*, while induced by XIP in both UA159 and the Δ*relA* mutant, did not differ between strains (Supplementary Figure S4B). Additionally, expression of *comD* and *cipB* were monitored and no differences were detected between UA159 and the Δ*relA* strains in cells treated only with DMSO. However, when the Δ*relA* strain was treated with sXIP there was no induction of *comD* and a lower level of induction of *cipB*, compared to that seen in the WT strain (Supplementary Figures S4C,D). Thus, the data are most consistent with the influences of Rel on competence arising from alterations in the expression of *comR*, although other inputs outside the ComRS–XIP system may modulate gene expression, sensitivity to XIP, and competence development.

To examine if the changes in *comR* mRNA levels were correlated with increased *comR* promoter activity, P*comR*, was fused to a *Streptococcus salivarius lacZ* gene and integrated in single copy into the chromosome of *S. mutans* at the *mtlA-phn* locus (Son et al., 2012; Guo et al., 2014). A significant decrease in LacZ activity was measured in the Δ*relA* background, compared to the WT strain (**Figure 4C**), with or without addition of sXIP. A similar reduction in promoter activity in the *relA*-deficient strain was also observed using a P*comX*-*lacZ* fusion (**Figure 4D**). Thus, deletion of *relA* can influence P*comR* activity, with the down-regulation of *comR* likely accounting for diminished activation of P*comX*.

### Complementation Analysis of the Δ*relA* Mutant

**FIGURE 5:**
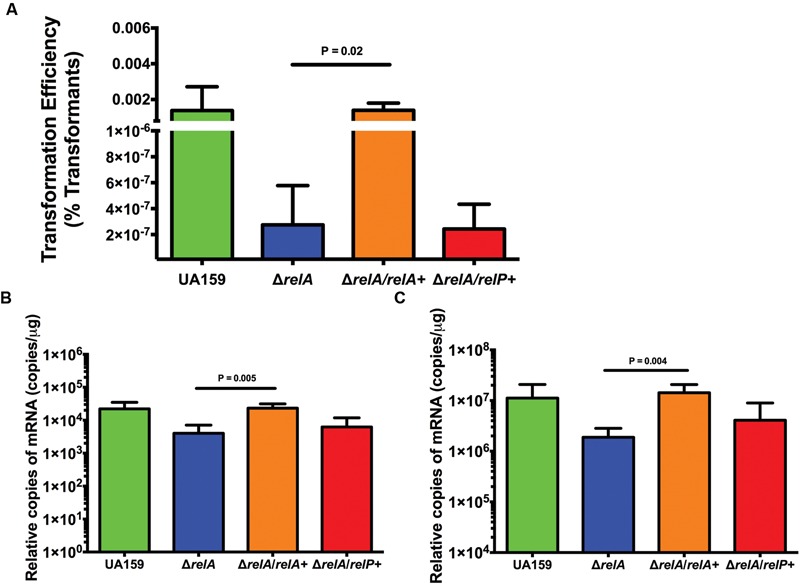
**Complementation of the Δ*relA* mutant with *relA* restores competence phenotypes. (A)** Transformation efficiency between strains UA159 (green bar), Δ*relA* (blue bar), *relA+*/Δ*relA* (*relA* mutant complemented with *relA*; orange bar), and *relP+*/Δ*relA* (*relA* mutant complemented with the gene for the small SAS RelP; red bar) in FMC medium with addition of 2 μM sXIP. sXIP and transforming DNA plasmid pDL278 (Sp^R^) were added when OD_600__nm_ reached 0.2. Serial dilutions of cultures were plated on BHI agar. Transformation efficiency was calculated by dividing the number of transformants by the total number of viable bacteria, then multiplying by 100. Data represent the average of three individual experiments conducted in triplicate. Statistical analysis was performed by Student’s *t*-test. **(B,C)** Measurement of mRNA by qRT-PCR of *comR* **(B)** or *comX* **(C)** in UA159 (green bars), Δ*relA* (blue bars), *relA+*/Δ*relA* (orange bars), and *relP+*/Δ*relA* (red bars) after addition of 2 μM sXIP. sXIP was added when OD_600__nm_ reached 0.2. After 1 h of incubation, cells were harvested by centrifugation, RNA isolated, and qRT-PCR performed. Gene expression was normalized to 16S rRNA expression. Data are derived from three biological replicates assayed in triplicate. Statistical analysis was performed by Student’s *t*-test.

Previously, a complemented Δ*relA* strain was constructed by providing an intact copy of *relA* gene in *trans* on plasmid pMSP3535, which allows for induction of the gene with subinhibitory concentrations of nisin (Lemos et al., 2004). To confirm that reductions in transformation efficiency and *com* gene expression were due to the loss of the Rel enzyme, the relevant phenotypes of the complemented strain (Δ*relA/relA^+^*) were compared with those of the *relA* mutant. The Δ*relA/relA^+^* strain displayed similar transformation efficiency as the WT strain (1.4 × 10^-3^ versus 1.5 × 10^-3^, respectively), whereas a strain in which the *relP* gene was provided on a plasmid in the *relA* mutant background (Δ*relA/relP^+^*) displayed transformation efficiencies equivalent to the Δ*relA* strain (2.8 × 10^-7^ to 2.4 × 10^-7^, respectively; **Figure 5A**). qRT-PCR of selected *com* genes in the complemented strains revealed that the Δ*relA/relA^+^* strain had similar quantities of mRNA as the WT strain for *comR* (**Figure 5B**) and *comX* (**Figure 5C**), whereas *com* gene expression in the Δ*relA/relP^+^* strain remained comparable to that in the Δ*relA* mutant. Thus, it was not sufficient to provide a plasmid-borne copy of a gene encoding only (p)ppGpp synthase activity (*relP*) in the *relA* mutant to achieve full complementation of RelA deficiency. Rather complementation of the RelA deficit was only achieved with an RSH enzyme containing both (p)ppGpp synthase and hydrolase activities (*relA*).

**FIGURE 6:**
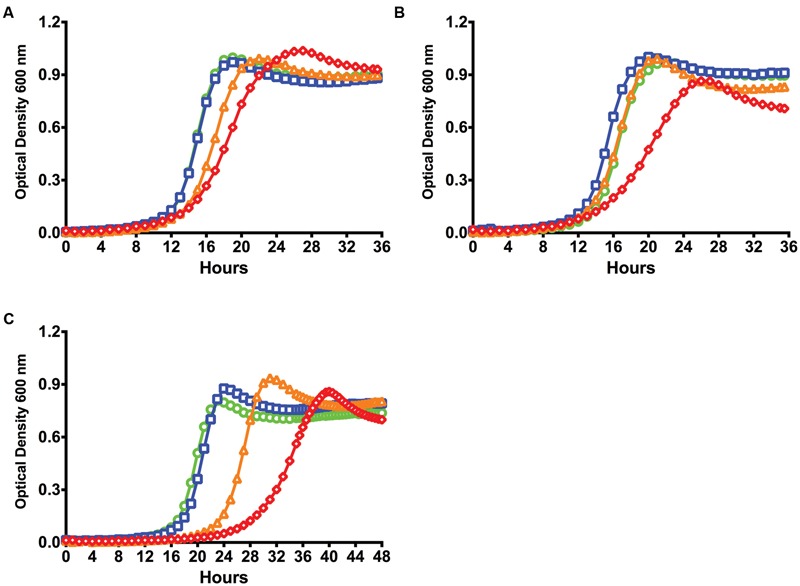
**Overexpression of *comR* and *comX* in a Δ*relA* genetic background overexpressing selected genes.** Growth kinetics of strains **(A)** 184ComS/Δ*relA*, **(B)** 184ComR/Δ*relA*, and **(C)** 184ComX/Δ*relA* with treatment of differing concentrations of sXIP (see text for more detail). Differing concentrations of sXIP were added to the culture medium: control of 1% DMSO (●; green), or 0.2 μM (■; blue), 2 μM (▲; orange), or 20 μM (◆; red) sXIP.

While internal accumulation of CipB induced by CSP via the ComDE system is thought to be involved in altruistic cell death and fratricide in a sub-population of cells (Perry et al., 2009), XIP-mediated cell death in chemically defined medium requires the ComRS system (Wenderska et al., 2012). This is apparently attributable to a recently discovered feedback circuit in which ComX binds to the *comE* promoter and leads to enhanced activity of the ComE regulon (Reck et al., 2015; Son et al., 2015b). Since expression of *comR* was decreased in the Δ*relA* strain, deficiencies in activation of *comX* by the ComRS system could be responsible for the observed changes in apparent growth inhibition by sXIP and transformation efficiencies. To explore this possible mechanistic linkage, we created strains that overexpressed *comS, comR*, and *comX* under the control of the P_23_ promoter on the shuttle plasmid pIB184 (Biswas et al., 2008) in the Δ*relA* genetic background. Growth kinetic studies in response to sXIP were conducted and compared to the Δ*relA* strain carrying only the pIB184 vector. Overexpression of *comS* elicited no changes in growth inhibition by sXIP (**Figure 6A**). Overexpression of *comR* in the *relA* mutant strain (184ComR/Δ*relA*) lead to modest growth inhibition in the presence of 2 μM sXIP, with an increase in the doubling time to 109 ± 4 min and reduction in final OD to 0.83 ± 0.05 (**Figure 6B**). Greater inhibition was observed in the presence of 20 μM sXIP, with an increased lag time (3 h), a doubling time of 194 ± 8 min, and a decrease in final yield to 0.71 ± 0.06. The 184ComX/Δ*relA* strain (overexpressing *comX*) showed the greatest growth inhibition in the presence of sXIP, with lag times of 24 and 29 h in the presence of 2 or 20 μM XIP, respectively (**Figure 6C**). Growth of the 184ComX/Δ*relA* strain in 20 μM sXIP resulted in doubling times of 240 ± 4 min and a final yield of 0.70 ± 0.04 after 48 h, very close to that observed for the WT strain. Thus, the loss of sensitivity of the strain lacking the RelA (RSH) enzyme to sXIP is clearly due to the aberrant decrease in *comX* mRNA levels and ComX production and can be compensated by overexpression of *comR* and *comX*, but not of *comS*. These results also suggest that the competence deficiencies of Δ*relA* stem from a lack of ComR, with enough ComR–XIP complexes present to fully saturate expression from the P*comS* promoter, but not the P*comX* promoter under similar conditions leading to deviations in transformation efficiency and cell lysis through ComX-dependent mechanisms.

### The Hydrolase Activity of RelA Is Essential for Effects on *com* Gene Expression

**FIGURE 7:**
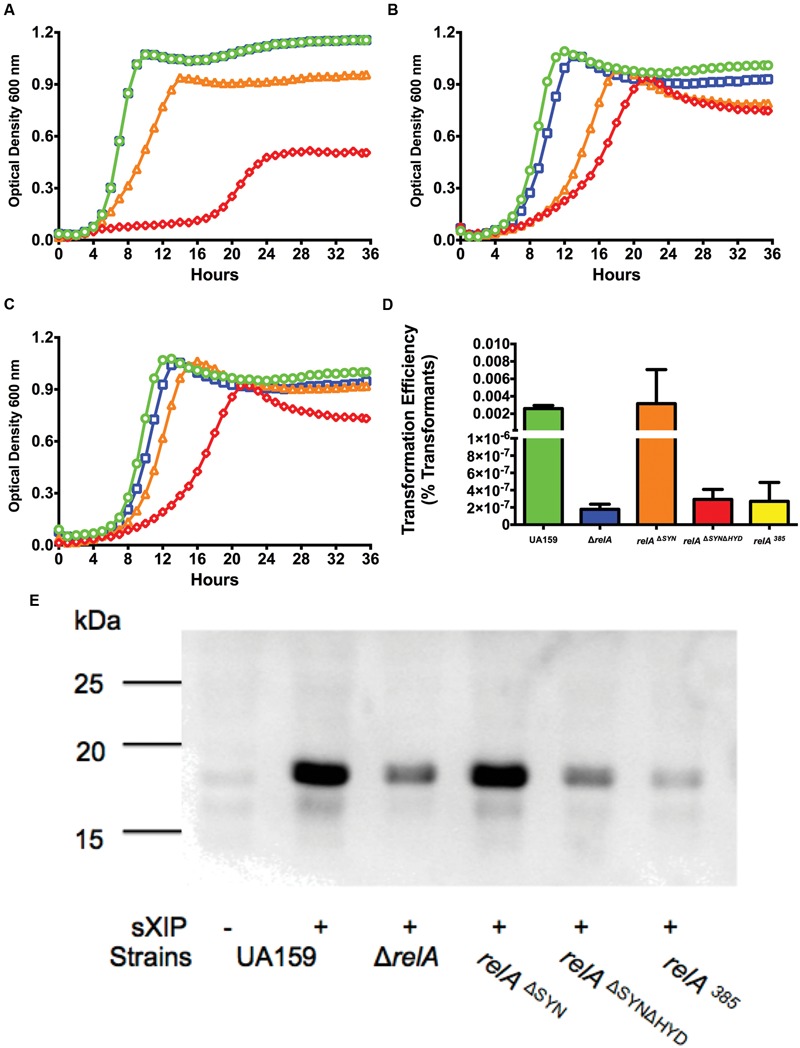
**RelA hydrolase activity is critical for *com* gene expression.** Growth curves of strains **(A)** *relA*^Δ^*^SY N^*, **(B)** *relA*^Δ^*^SY N^*^Δ^*^HY D^*, and **(C)** *relA^385^* treated with different concentrations of sXIP. Bacterial cultures were grown in the chemically defined medium FMC containing 1% DMSO (●; green), or 0.2 μM (■; blue), 2 μM (▲; orange), or 20 μM (◆; red) sXIP. **(D)** Transformation efficiency of UA159 (green bar), Δ*relA* (blue bar), *relA^ΔSY N^* (orange bar), *relA^ΔSY N^*^Δ^*^HY D^* (red bar), and *relA^385^* (yellow bar) in FMC medium after treatment with 2 μM sXIP. sXIP and purified pDL278 (Sp^R^) were added when OD_600__nm_ reached 0.2. Cultures were diluted and plated, and transformation efficiency was calculated as detailed in **Figure 3**. Data are the average of three individual experiments conducted in triplicate. **(E)** Detection of ComX in the *relA* mutant grown in FMC with either 1% DMSO (-) or 2 μM sXIP (+) added at OD_600__nm_ = 0.2 and then incubated for 1 h. ComX was detected as above, using the same molecular mass standards (in kDa).

The RelA enzyme can synthesize and degrade (p)ppGpp, with the catalytic domains for these activities located in the N-terminal portion of the enzyme. Synthetase-ON/hydrolase-OFF and synthetase-OFF/hydrolase-ON states are controlled by allosteric regulation mediated by the C-terminal domain (Mechold et al., 1996, 2002). Analysis of the crystal structure of the N-terminal fragment (residues 1–385) of the Rel enzyme of *Streptococcus dysgalactiae* subsp. *equisimilis* (Rel*_Seq_*) revealed amino acid residues critical for the activities of both domains, with mutation of specific amino acids leading to loss of activity in one domain without affecting the function of the other (Hogg et al., 2004). RelA of *S. mutans* shares 85% identity at the amino acid level with Rel*_Seq_*, and residues found to play critical roles in the catalytic activity of Rel*_Seq_* are conserved in *S. mutans* (data not shown). Amino acid substitution mutations in the Rel*_Seq_* synthetase and hydrolase domains were recapitulated in the *S. mutans* RelA enzyme to determine if one or both enzymatic activities were critical for normal *com* gene expression. Inhibition of the synthetase function was achieved by mutating the aspartate residue at position 264 to glycine (D264G, *relA^ΔSY N^*; Hogg et al., 2004). When grown with increasing concentrations of sXIP, this strain displayed growth inhibition similar to the WT strain (**Figure 7A**). Mutation of the catalytic site of the hydrolase domain was achieved by substituting a threonine residue with proline at position 151 (T151P; Hogg et al., 2004). Interestingly, viable colonies containing this single substitution could not be recovered, and DNA sequence analysis of transformants that did contain the desired mutation were found to have secondary mutations in the *relA* gene; either an additional mutation in the synthetase domain or a frameshift mutation that led to production of a truncated enzyme that was predicted to be non-functional. Thus, we were unable to obtain a *relA^ΔHY D^* strain. These results suggest that strains of *S. mutans* in which the loss of function of the RelA (p)ppGpp hydrolase domain has occurred without concurrent loss of the synthetase domain are non-viable. Notably, the *relA^ΔHY D^* mutation could also not be obtained in a strain lacking *relPQ*, so synthesis of (p)ppGpp by RelA and the associated over accumulation of alarmone in the absence of the hydrolase domain appears sufficient to account for lack of growth of an otherwise-WT *relA^ΔHY D^* mutant. We were, however, able to incorporate the T151P mutation into the *relA^ΔSY N^* strain to create a viable strain expressing a mutant derivative of RelA with no (p)ppGpp synthase or hydrolase activity (*relA^ΔSY NΔHY D^*). When *relA^ΔSY NΔHY D^* strain was evaluated for its sensitivity to XIP (**Figure 7B**), the strain displayed a level of resistance to XIP similar to the Δ*relA* mutant, adding further support that loss of the hydrolase domain is responsible for the changes in XIP sensitivity. These observations are consistent with the previous findings (**Figure 5**) that addition of a synthetase only (*relP*) back to the Δ*relA* strain is unable to complement the reduction in competence activity phenotypes. To further confirm the importance of the hydrolase domain in responses of *S. mutans* to XIP, we examined a strain (*relA^385^*) that includes an insertion of an antibiotic resistance cassette beginning at amino acid 386, resulting in a 385-aa peptide containing only the N-terminal domain of RelA. This strain produces high basal levels of (p)ppGpp (data not shown), with the produced peptide containing no detectable (p)ppGpp degrading activity (Mechold et al., 2002). The *relA^385^* strain showed growth kinetics in the presence of increasing concentrations of sXIP similar to the Δ*relA* and *relA^ΔSY NΔHY D^* strains (**Figure 7C**). Transformation efficiency (**Figure 7D**) and ComX levels in each of these strains was consistent with the growth characteristics in the presence of XIP (**Figure 7E**). Taken together, these results show that the hydrolase domain of RelA plays an essential role in optimizing (p)ppGpp levels to allow for normal responses to XIP and expression of *com* genes.

### Loss of RelA Results in Changes to the Phenotypes of Δ*rcrR* Strains

**FIGURE 8:**
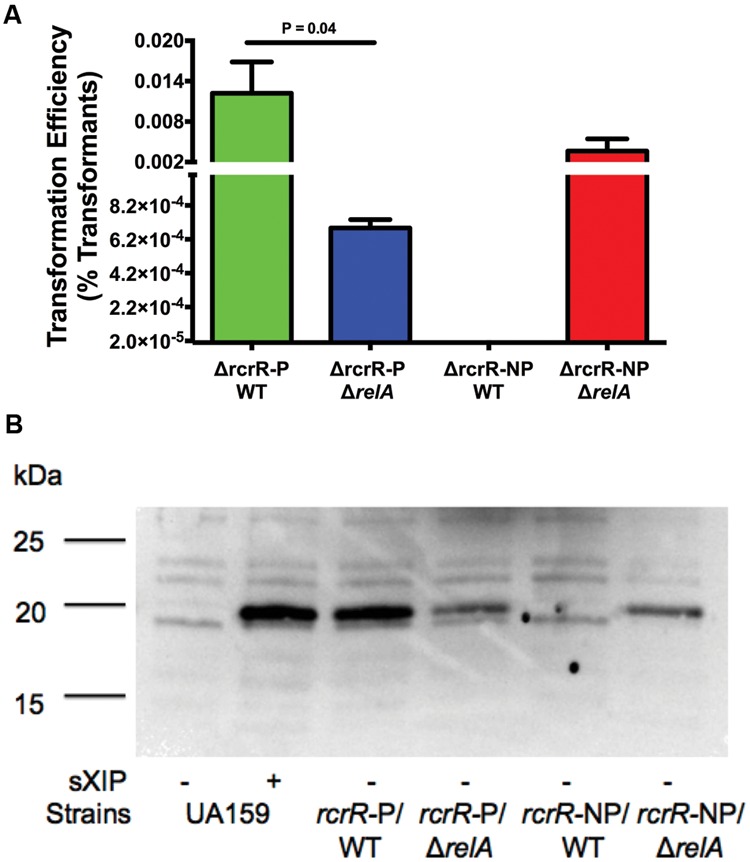
**Effects of RelA on the phenotypes of Δ*rcrR* stains. (A)** Transformation efficiency of strains Δ*rcrR*-P (green bar), Δ*rcrR*-PΔ*relA* (blue bar), Δ*rcrR*-NP (no bar because it is completely non-transformable), and Δ*rcrR*-NPΔ*relA* (red bar) in FMC medium with addition of 2 μM sXIP. sXIP and transforming DNA plasmid pDL278 (Sp^R^) were added when OD_600__nm_ = 0.2. Cultures were plated and transformation efficiency was determined as above. Data are the average of three individual experiments conducted in triplicate. **(B)** Detection of ComX in FMC with either 1% DMSO (-) or 2 μM sXIP (+) added at OD_600__nm_ = 0.2 and then incubated for 1 h. ComX was detected as above with the same molecular mass standards (in kDa).

Two genes (*tpx, cipI*) separate the *rcrRPQ* and *relPRS* operon, all transcribed in the same direction. The products of *rcrRPQ* and *relP* profoundly and concurrently impact stress tolerance, (p)ppGpp production, and genetic competence in *S. mutans* (Seaton et al., 2011). Moreover, the MarR-type regulator RcrR was found to interact with the *relP* promoter *in vitro* (Seaton et al., 2015) and the levels of the ABC transporters RcrP and RcrQ and two small peptides encoded at the 3′ end of *rcrQ* exert dominant control over competence development (Ahn et al., 2014). Of particular relevance here, a Δ*rcrR*-P (polar) mutant is hyper-transformable, whereas a Δ*rcrR*-NP (non-polar) mutant is completely non-transformable, in part due to lack of expression of full-length *comX* mRNA and production of very low levels of ComX (Kaspar et al., 2015). As the phenotypes displayed by the RcrRPQ operon and (p)ppGpp are intertwined, we reasoned that RcrRPQ and its effectors could be a source for modulation of (p)ppGpp accumulation leading to changes in the transformable state of cells based on environmental signals/conditions. The extremely disparate competence phenotypes displayed by the Δ*rcrR* polar and non-polar strains provided us the opportunity to explore mechanisms related to RelA activity and its role in *com* gene expression, which would have been more difficult to analyze in the WT background. Hence, we examined the effects of deleting *relA* (Δ*relA*) in the *rcrR* mutants. The transformation efficiency of the Δ*rcrR-*PΔ*relA* double mutant (**Figure 8A**) had reduced transformation compared with the Δ*rcrR*-P mutant (6.8 × 10^-4^ versus 1.2 × 10^-2^, respectively). A decrease in ComX protein abundance was also observed in this double mutant (**Figure 8B**). Interestingly, a complete reversal of the non-transformable phenotype of the Δ*rcrR*-NP strain was evident when the Δ*relA* mutation was present in the same strain. In particular, the Δ*rcrR*-NP strain alone could not be transformed, but the Δ*rcrR*-NPΔ*relA* double mutant displayed transformation efficiency comparable to the hyper-transformable Δ*rcrR*-P strain (1.3 × 10^-2^), and ComX levels were also restored to a level similar to those expressed in the Δ*rcrR*-P strain. Collectively, these data provide further confirmation of the critically important roles of the RelA enzyme and (p)ppGpp in modulation of genetic competence and peptide signaling behaviors in *S. mutans*.

## Discussion

Many of the organisms that make up the microbiome of the oral cavity do not survive outside of their host (Lemos et al., 2013). These bacteria have adapted to thrive in specific habitats in the oral cavity and employ substantial phenotypic plasticity to optimize growth in their continually fluctuating environments. To achieve this, the organisms utilize complex regulatory networks to control gene expression in concert with allosteric modulation of enzyme activities to respond to numerous stimuli. From a variety of studies, it appears that *S. mutans* has evolved in a way that it primarily utilizes a set of global transcriptional regulators, conserved stress-response pathways, monitoring of metabolic status, and carbohydrate transporters as the dominant mechanisms to interpret its environment and make adjustments to its gene expression patterns, physiologic status, and virulence capacities. In this study, we investigated and discovered a novel linkage in *S. mutans* of gene products that participate in the stringent response and control of growth rate (Lemos et al., 2007) via modulation of (p)ppGpp levels with the regulatory network for genetic competence for natural transformation; the latter benefitting the organism from a nutritional standpoint and by enhancing its genomic diversity.

From the data presented here, we can begin to develop a working model for the connection of (p)ppGpp to the development of genetic competence. In this model, (p)ppGpp concentrations within individual cells within a population serves as a source for “noise” (Lidstrom and Konopka, 2010), creating variance through fluctuations in *com* gene expression, with *comR* promoter activity acting as a primary control point (**Figure 4C**). A similar type of regulatory switch has been documented in *E. coli* (Maisonneuve et al., 2013), where variations in (p)ppGpp pools within a population lead to a stochastic response in expression of toxin–antitoxin modules, giving rise to a persister population due to growth arrest. A similar phenomenon may govern competence and stress tolerance behaviors in *S. mutans*, where individual cells must choose to commit to competence development through activation of the *comRS* pathway, and ultimately activate *comX* expression, or remain non-competent. A bimodal switch (sub-population response) for *comX* expression is observed in *S. mutans* cells growing in a chemically complex medium such as BHI, with proposed autofeedback regulation occurring at the level of ComRS (Son et al., 2012). Through use of promoter fusion strains and quantification of mRNAs and ComX protein, we show here that down-regulation in P*comR* activity in a *relA* mutant can cause a significant drop in *comR* mRNA, with an associated decrease in ComX levels. These changes should have a direct effect on transformation efficiency of the population, as well as on the proportion of cells that may display growth inhibition or undergo lysis after exposure to peptide signals, consistent with results presented in this communication.

We also propose that an “optimal window” exists for intracellular (p)ppGpp concentrations that allows cells to proceed into the competent state via activation of *com* gene expression. The existence of such a “window” can be seen in the different behaviors of *rel* mutant strains, primarily with a *relA* mutant that accumulates higher basal levels of (p)ppGpp due to loss of hydrolase activity, and a strain lacking the SAS enzymes (Δ*relPQ*), which accumulates low basal levels of (p)ppGpp. The *relA* mutant strain showed a high degree of resistance to the growth inhibition by XIP (**Figure 3A**), whereas the Δ*relPQ* double mutant was very sensitive to 2 μM sXIP (**Figure 3B**). Thus, the internal concentrations of (p)ppGpp correlate with growth sensitivity to the sXIP peptide, with higher basal levels of (p)ppGpp favoring resistance to the signal and lower levels of (p)ppGpp leading to an increased response. Once outside of the optimal window, cells could potentially change their behavior toward the signal altogether. Such behavior is apparent in the (p)ppGpp^0^ strain, which is devoid of alarmone and is completely resistant to the apparent growth inhibitory effects of sXIP (**Figure 1B**). Additionally, after cells reach a high concentration threshold of (p)ppGpp accumulation, *com* gene expression can be effected. One must also take into account the growth rates of the strains in medium absent XIP to account for their observed behaviors in the presence of sXIP peptide. Specifically, the Δ*relA* strain displays a doubling time that is 40 min greater than the WT strain in FMC medium alone, likely due to accumulation of (p)ppGpp in the absence of the hydrolase activity of RelA, whereas *relP* and/or *relQ* mutant strains have doubling times comparable to UA159 (Lemos et al., 2007). Thus, the changes in the behavior of the *rel* mutant strains in response to exogenous XIP is even more profound than if the growth rates of the *relA* mutant was more similar to those of *relP* and *relQ* mutants.

**FIGURE 9:**
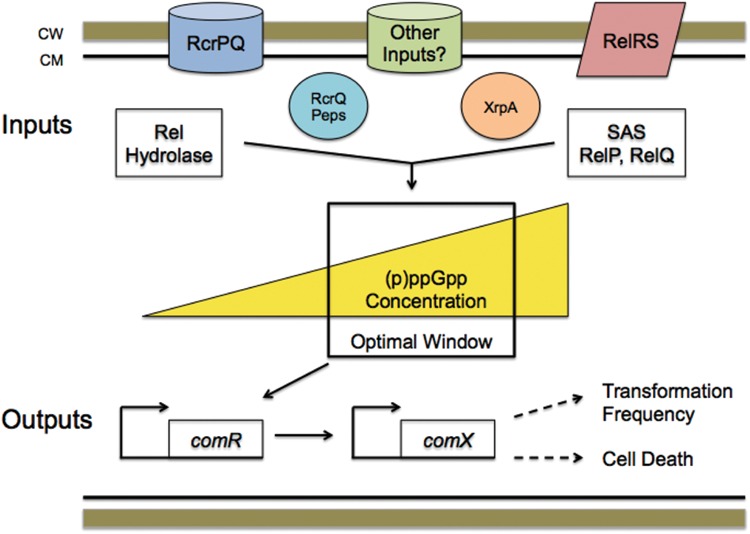
**Working model integrating (p)ppGpp levels with the development of competence in *S. mutans*.** The model predicts that (p)ppGpp levels play an essential role in determining cell fate after competence gene activation. Numerous inputs that monitor environmental conditions either through cell density or by serving as stress sensors, including RelRS, RcrPQ, and others, e.g., CiaRH and ScnRK, along with the RcrQ peptides (labeled “peps”) and XrpA, all modulate internal (p)ppGpp accumulation via the activity of the RelA hydrolase or the small alarmone synthetases RelP and RelQ. (p)ppGpp fluctuations then serve as a source of “noise” or stochasticity that drives heterogeneity within the population. The internal (p)ppGpp concentration influences P*comR* activity, directly or indirectly, with consequential influences on P*comX* promoter activity. An “optimal window” in (p)ppGpp concentrations is necessary to achieve “normal” *com* gene expression responses to peptides. (p)ppGpp levels less than or greater than those in the “optimal window” result in changes in responses to signal inputs that alter *com* gene expression. Although not shown, ComX stability is also a determining factor for competence and cell death. Thus, shifts in ComX levels and activity, which are controlled transcriptionally and post-transcriptionally, dictate the ratio of cells undergoing transformation or cell death within a population of cells. In this way, (p)ppGpp levels modulated by RelA hydrolase activity allow cells to integrate physiologic status with commitments to signal response, progression to the competent state, or cell death. Other factors, like toxin:antitoxin modules, likely contribute to this decision network in a manner that also depends on (p)ppGpp levels.

One aspect of our working model (**Figure 9**) that is not yet well developed is how RcrRPQ and the effector peptides encoded at the end of *relQ* influence (p)ppGpp pools and, ultimately, competence development. The *rcrRPQ* operon and (p)ppGpp are tightly connected in their regulation, as previously shown by Seaton et al. (2011). In their study, mutation of the SAS-encoding gene *relP* resulted in changes in *rcrR* promoter activity and *rcrRPQ* expression, which ultimately impacted the expression levels of the RcrPQ ABC-transporters and peptides encoded at the end of *rcrQ* (Ahn et al., 2014). Importantly, it has been shown that very small fluctuations in the activity of the *rcrR* promoter or levels of *rcrRPQ* transcripts can have profound effects on competence-related behaviors (Seaton et al., 2015). While the exact mechanism for how (p)ppGpp and RcrRPQ interact to exert these effects on competence have yet to be disclosed, the significance of this connection is strengthened by data presented here showing that deletion of *relA* in a Δ*rcrR*-NP strain completely reverses the non-transformable phenotype (**Figure 8**). We found previously that the non-transformability of Δ*rcrR*-NP is accompanied by, and largely attributable to, the loss of a full-length *comX* mRNA and ComX protein along with activation of expression of an ORF intragenic to *comX* termed XrpA (Kaspar et al., 2015). When point mutations are introduced to prevent production of XrpA while keeping *comX* intact in a Δ*rcrR*-NP background, full-length *comX* expression is restored, ComX protein reappears and transformability is on par with the *rcrR-*P hyper-transformable strain (Kaspar et al., 2015). Interestingly, virtually identical changes occur when *relA* is deleted or RelA enzymatic activity is ablated in the Δ*rcrR*-NP background. Thus, control of (p)ppGpp levels by the RelA hydrolase play a key role in allowing the cell to determine whether a full-length *comX* transcript can be produced or whether the transcript will be processed and high levels of the *xrpA-*specific transcript produced. While the exact mechanism by which (p)ppGpp levels influence this decision remain to be established, the known roles of these alarmones in cellular physiology and gene expression provide evidence that the cells can use (p)ppGpp to integrate decisions to become competent or to select programmed cell death pathways with assessment of the physiologic status of individual cells. In this context, further exploration of how (p)ppGpp levels influence *comR* transcription, whether (p)ppGpp is an allosteric regulator of RcrR, and if there are interactions between alarmone and the RcrPQ transporters and effector peptides is planned.

We were also able to detect changes in (p)ppGpp accumulation after addition of sXIP to cultures of *S. mutans*. These changes could be observed 40 min after sXIP addition, and with the greatest effects coming with greater than 1 μM sXIP added to the cultures (**Figure 2**). It is known that *S. mutans* displays the greatest growth inhibitory defects in the presence of >1 μM sXIP peptide (Wenderska et al., 2012). One could thus propose that the reduced growth rate exhibited by *S. mutans* in response to sXIP at these concentrations could be due, at least in part, to the increased accumulation of (p)ppGpp slowing the overall growth rate of the organism.

In *Bacillus subtilis*, the ComGA protein, an apparent ortholog of ComYA in *S. mutans*, participates in growth arrest during the K-state by inhibiting replisome assembly in a (p)ppGpp-independent manner. However, ComGA can also interact directly with RelA to increase the pool of (p)ppGpp in cells, inhibiting replication and rRNA synthesis (Haijema et al., 2001; Hahn et al., 2015). The coupling of growth arrest to transformation may allow for cells to overcome the fitness burdens of production of competence proteins and internalization of DNA, while concurrently creating sufficient opportunity for repair of the genome associated with recombination events that occur after DNA uptake (Briley Jr et al., 2011). In this study, we found the increase of (p)ppGpp accumulation upon addition of sXIP is most likely catalyzed by either RelP or RelQ, and not by RelA. When measured by real-time PCR, we were able to detect a 1.89-fold increase in *relQ* expression, with a concurrent 2.79-fold decrease in *relA*, whereas *relP* mRNA levels remained unchanged (**Figure 2B**). Instead, we measured a significant increase in *relQ* expression. One possible explanation for the observed changes in *relQ* expression is that it is co-transcribed with the gene (*pta*) for phosphotransacetylase, which provides acetyl phosphate that can be used for ATP generation (Kim et al., 2015). It is reasonable to expect that the stress induced by exposure to high levels of XIP could alter metabolism in a way that directs cells to slow growth via RelQ-dependent (p)ppGpp synthesis, while shifting from homofermentative metabolism to acetate production, which yields an additional ATP. And, as proposed above, fluctuations in (p)ppGpp pools as a result of physiologic adaptations when signal molecules are present may help to drive stochastic events that direct segregation of cells into subpopulations that maintain normal growth, undergo autolysis or commit to late competence gene expression (Lemme et al., 2011).

The competence development pathways of *S. mutans* have been shown to be unusually complex, with additional layers of complexity of the system being revealed as more mutants are evaluated and environmental variables are explored (Ahn et al., 2014; Guo et al., 2014; Kaspar et al., 2015; Son et al., 2015a). Perhaps, though, it is not surprising that the spectrum of variables and inputs governing the competence system, which include RcrRPQ, multiple unique peptides (XrpA, *rcrQ-*associated peptides) and three (p)ppGpp synthases, is so elaborate in this opportunistic pathogen. Were *S. mutans* to engage in the activation of the competence cascade in response to peptide signals, with the associated expense of energy to produce and deploy the machinery for DNA uptake and processing, irrespective of physiologic status of the cells, then the fitness of the population could be severely compromised. Instead, a picture is emerging that (p)ppGpp serves a critical function in allowing the cells to distill a diverse set of physiologic parameters (nutrient availability, pH, redox, protein, or envelope damage) when signal peptides reach some critical level to govern genetic responses at the single cell level in a way that is optimal for survival of individual cells and beneficial for the local population of *S. mutans*. It is also notable that the virulence of *S. mutans* is so strongly tied to its physiology and metabolism; i.e., damage to the tooth requires acid production through glycolysis and an elaborate regulon that allows for constitutive and adaptive acid tolerance. Perhaps this is why the stress tolerance regulon and genetic competence in this organism are so tightly tied to the organism’s ability to express critical virulence related phenotypes (biofilm formation, acid and oxidative stress tolerance). Thus, dissection of the regulatory network described here, which consists of many effector molecules (e.g., XrpA) that appear to be unique to *S. mutans*, has tremendous potential for guiding the development of strategies to reduce the incidence of disease caused by this opportunistic pathogen. Future studies will be targeted at how (p)ppGpp, peptide effector molecules, and regulatory proteins interact to collect inputs and govern the behavior of individual cells and populations of *S. mutans.*

## Author Contributions

JK contributed to conception, design, data acquisition and analysis, data interpretation, drafted and critically revised the manuscript; JNK contributed to the design, data acquisition and analysis, data interpretation, and critically revised the manuscript; S-JA contributed to conception, design, data interpretation and critically revised the manuscript; RB contributed to conception, design, data interpretation, drafted and critically revised the manuscript. All authors gave final approval to the manuscript and agree to be accountable for all aspects of the work.

## Conflict of Interest Statement

The authors declare that the research was conducted in the absence of any commercial or financial relationships that could be construed as a potential conflict of interest.

## Footnotes

1 http://rsbweb.nih.gov/ij/
