# Supplementary material for: An Essential Role for (p)ppGpp in the Integration of Stress Tolerance, Peptide Signaling, and Competence Development in Streptococcus mutans
Source: Front Microbiol. 2016 Jul 28;7:1162. doi: 10.3389/fmicb.2016.01162 (PMC4963387; doi:10.3389/fmicb.2016.01162)
Supplement: Supplementary file 1 [file Presentation_1.PDF]

Table S1. Primers Used in Study

Primer	Nucleotide Sequence (5'-3')	Use
S. mutans Strain Construction		
RelA-5'	GGG GTT TCA AGG AGA ATG GAA CAA	Amplification of *relA* sequence
RelA-3'	GCC ATA ATA GTC TGA CGC AGT GGC	Amplification of *relA* sequence
SeqRelA-5'	GGT CTT GGG CTT ATA ATC ATA TCG	Sequencing of *relA* sequence
SeqRelA-3'	ATT GAA TGC ATC AAA TGG TCA CTG	Sequencing of *relA* sequence
RelA::D264G-A	GCC ACG GAA TGC CAT AGC GG	Arm 1 of *relA*$^{\Delta SYN}$ mutagenesis
RelA::D264G-B	CGA ATA GCG ATC AAA CCA TAA ATT TGA TC	Arm 1 of *relA*$^{\Delta SYN}$ mutagenesis
RelA::D264G-C	GAT CAA ATT TAT GGT TTG ATC GCT ATT CG	Arm 2 of *relA*$^{\Delta SYN}$ mutagenesis
RelA::D264G-D	GGC CAC ATA GCC CTG CTC	Arm 2 of *relA*$^{\Delta SYN}$ mutagenesis
RelA::D264G-Seq	CCC TTG GCG CAT CGG CTG GGG	Sequencing of *relA*$^{\Delta SYN}$ mutagenesis
RelA::T151P-A	GAT CAC TGT GGT TTA GCC TGC C	Arm 1 of *relA*$^{\Delta HYD}$ mutagenesis
RelA::T151P-B	CGT AAA TGC CTC AGC GGC CGC ATA TTA TG	Arm 1 of *relA*$^{\Delta HYD}$ mutagenesis
RelA::T151P-C	CAT AAT ATG CGG CCG CTG AGG CAT TTA CG	Arm 2 of *relA*$^{\Delta HYD}$ mutagenesis
RelA::T151P-D	GCC ATT GGA AGC GTC TTG C	Arm 2 of *relA*$^{\Delta HYD}$ mutagenesis
RelA::T151P-Seq	GAT GGT GTC ACA AAG CTA GGG	Sequencing of *relA*$^{\Delta HYD}$ mutagenesis
P*comR*-LacZ-SacI	GGA GAG CTC TCT CAT TAA CAA TCT C	Construction of PcomR-LacZ
P*comR*-LacZ-BamHI	CTT TGG ATC CAA AAC CTT TTC CTA TAA TCT CTG TC	Construction of PcomR-LacZ
RelP-BamHI-F	GAA GGA TCC TGT AAG AAG GAT GAA TTA TGT C	Construction of pIB184RelP
RelP-EcoRI-RV	GTT AAG AAT TCT TAT TCA CCA CTT CCT AC	Construction of pIB184RelP
qRT-PCR Primers		
ComX Sense	AAT AAG GGT AAG CCA ATT GTA TGG A	Expression of *comX*
ComX Antisense	TGG TGC AAA ATC AAC ATT CC	Expression of *comX*
ComR Sense	TAT TAC GAA GGC CAA CCT AT	Expression of *comR*
ComR Antisense	TTC TTC TTC AGG CAA ATC AT	Expression of *comR*
ComS Sense	TCA AAA AGA AAG GAG AAT AAC A	Expression of *comS*
ComS Antisense	TCA TCT GAG ATA AGG GCT GT	Expression of *comS*
ComYA Sense	ATT ATC TCT GAG GCA TCG TCC G	Expression of *comYA*
ComYA Antisense	ACC ATT GCC CCT GTA AGA CTT G	Expression of *comYA*
ComD Sense	TAT GGT CTC TGC CTG TTG C	Expression of *comD*
ComD Antisense	TGC TAC TGC CCA TTA CAA TTC C	Expression of *comD*
CipB Sense	GCG GAT GGA ATT GTG CAG CAG	Expression of *cipB*
CipB Antisense	TCC GAT TCC TCC AGC AAT AGC C	Expression of *cipB*
RcrR Sense	TGT TTT AAC GCC ATT AGG TCA GG	Expression of *rcrR*
RcrR Antisense	TCC GAG CAA CTG ATA AGT CTT CC	Expression of *rcrR*

*Underline denotes restriction enzyme site

Supplemental Figure 1A – *comX* mRNA

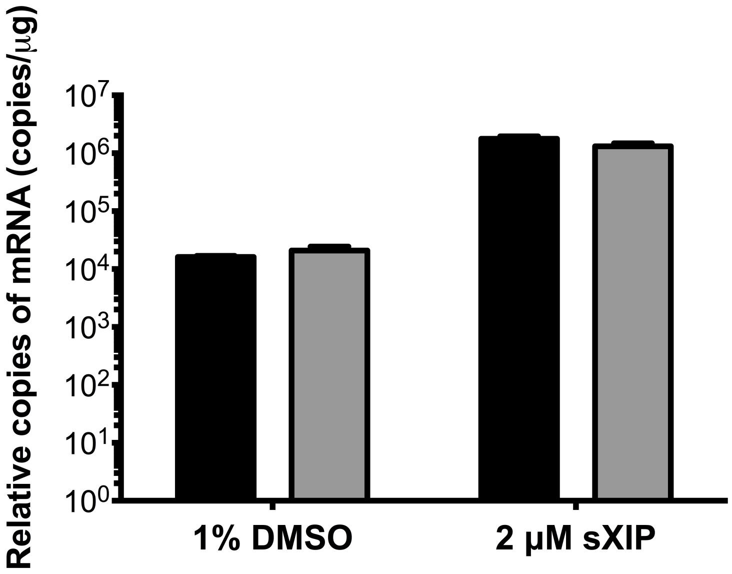

Supplemental Figure 1B -- *comYA* mRNA

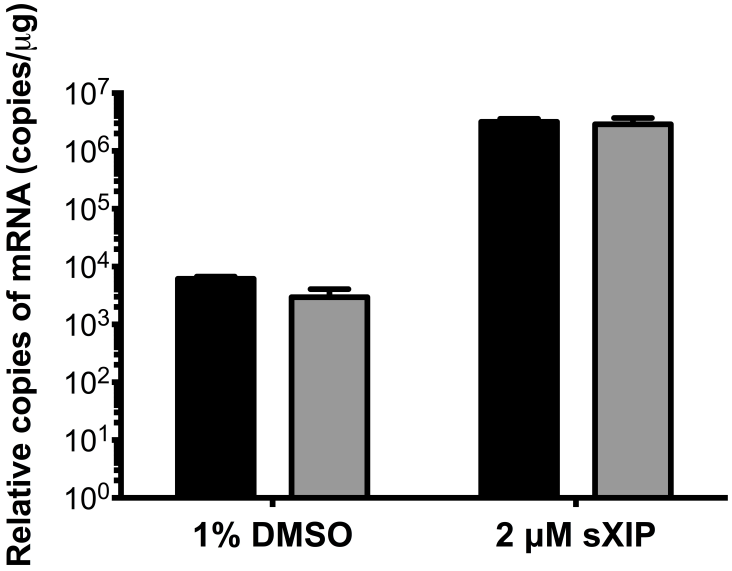

Supplemental Figure 1C

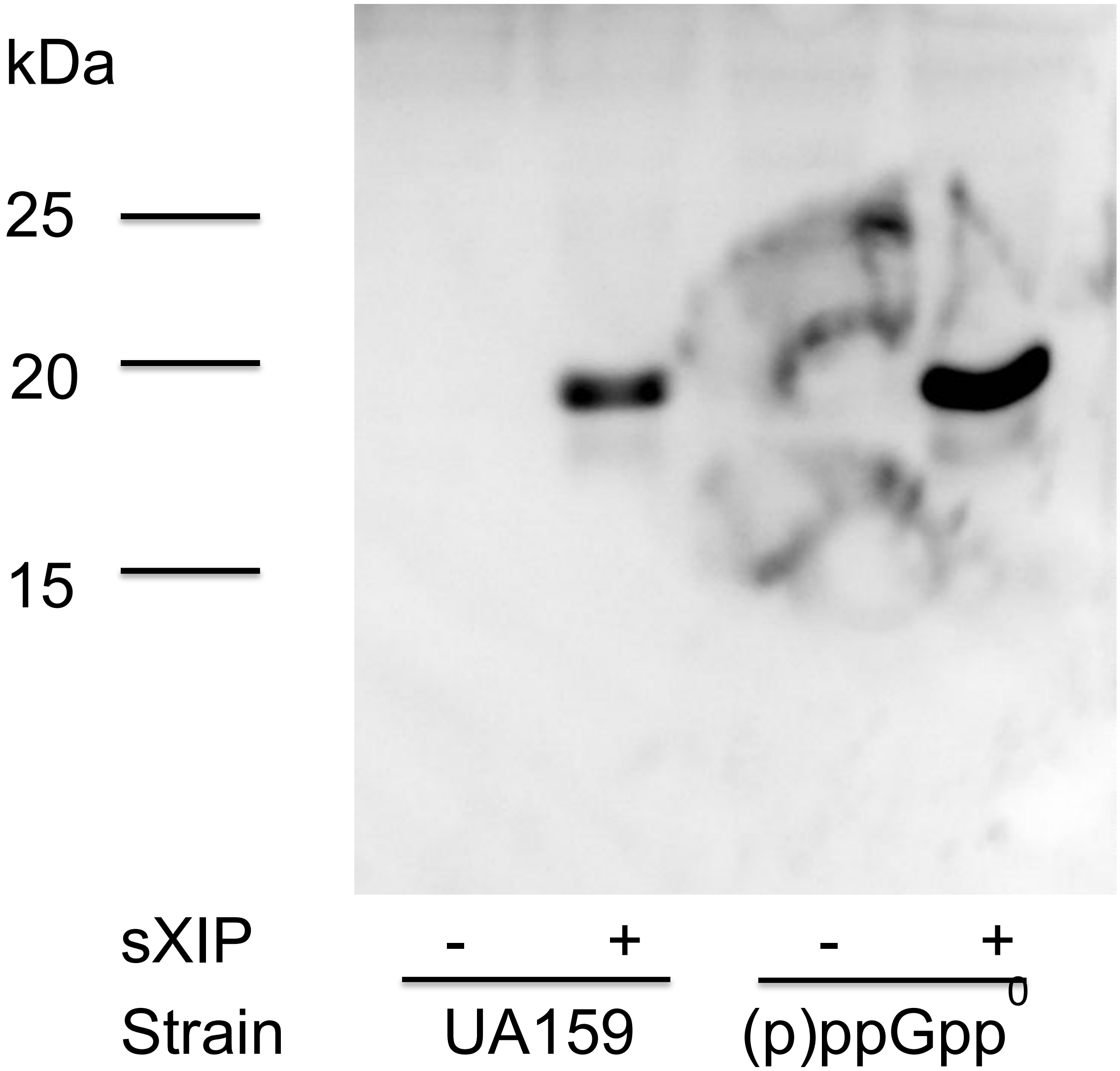

Supplemental Figure 1D – *comD* mRNA

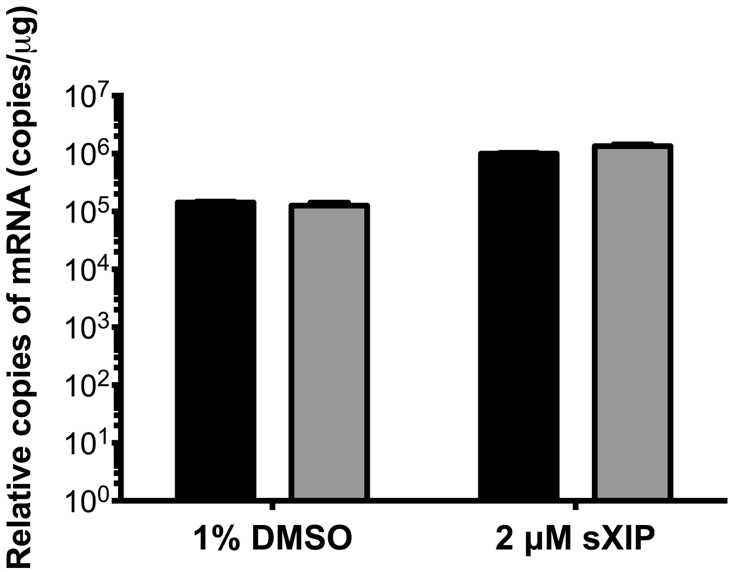

Supplemental Figure 1E – *comR* mRNA

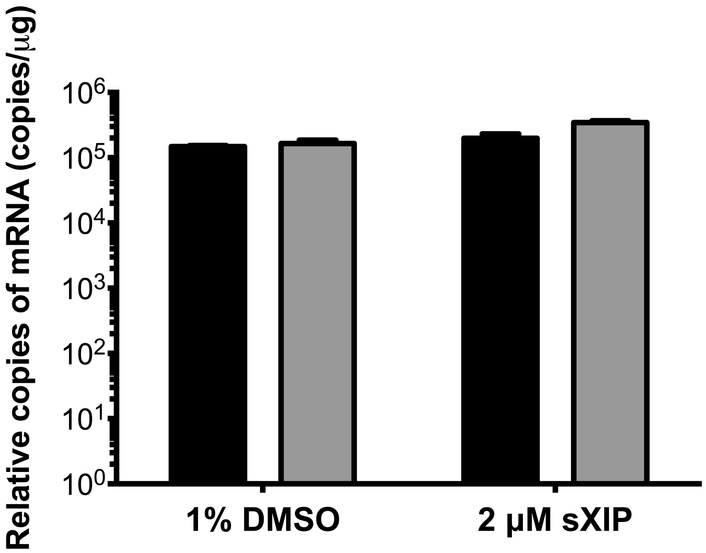

Supplemental Figure 1F – *comS* mRNA

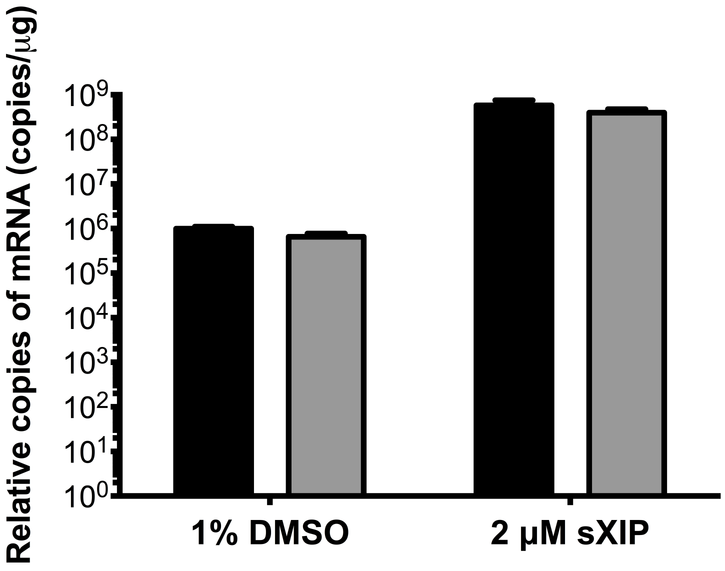

Supplemental Figure 1G – *rcrR* mRNA

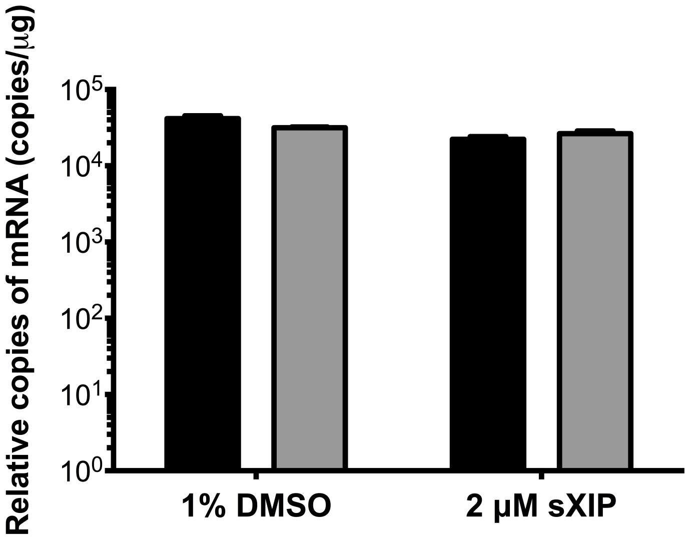

Fig. S1. Measurement of *com* gene expression in UA159 and the (p)ppGpp^0 strain.

Measurements of mRNA using qRT-PCR of (A) *comX*, (B) *comYA*, (D) *comD,* (E) *comR,* (F) *comS,* and (G) *rcrR* in *S. mutans* UA159 (black bars) and its (p)ppGpp^0 derivative (Δ*relAPQ*; gray bars) after addition of 2 μM sXIP. sXIP was added when OD_{600} reached 0.2. After one hour of incubation, cells were harvested by centrifugation, RNA isolated, and RT-qPCR was performed. Gene expression was normalized to 16S rRNA expression. The data represent three biological replicates and assays were performed in triplicate. (C) Detection of ComX in UA159 and the (p)ppGpp^0 strain in lysates from cells grown in FMC treated with either 1% DMSO (-) or 2 μM sXIP (+) at $OD_{600\ nm}$ = 0.2 and then incubated for 1 hour. ComX was detected using a 1:5000 dilution of primary antisera raised against full-length recombinant ComX from *S. mutans*. Molecular mass standards (in kDa) are shown to the left. The calculated molecular mass of ComX is 19 kDa.

Supplemental Figure 2

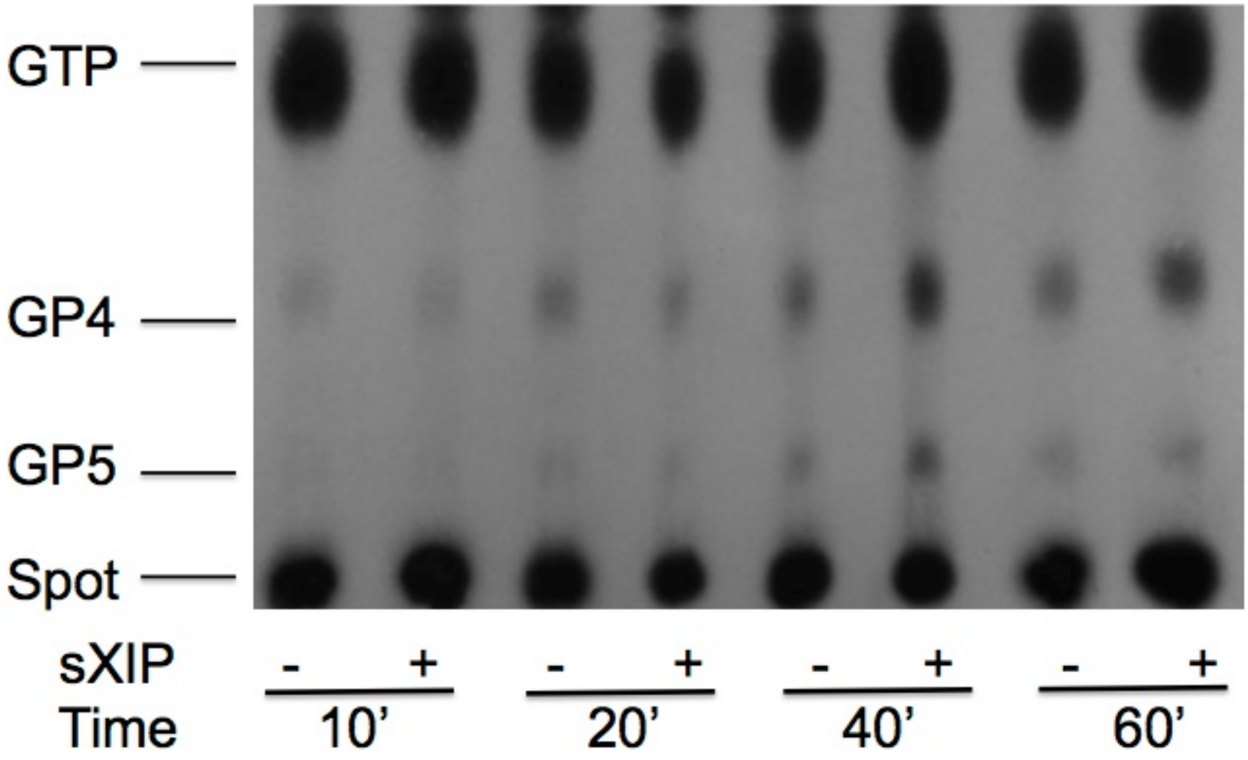

Fig. S2. Accumulation of (p)ppGpp in response to addition of sXIP. (p)ppGpp levels in UA159 over time after addition of either 1% DMSO (-) or 2 μM sXIP (+). Time denotes minutes after ^{32}P-orthophosphate and sXIP addition. Cells were labeled with ^{32}P-orthophosphate in FMC medium when $OD_{600\ nm}$ reached 0.2, along with addition of either 1% DMSO or 2 μM sXIP. Nucleotides were extracted by addition of 13 M formic acid, followed by three freeze-thaw cycles. Cells were removed by centrifugation and the resulting supernates were spotted onto PEI-cellulose plates for TLC in 1.5 M KH_2PO_4. Identity of the migrating nucleotides is shown to the left. (GP4 – ppGp; GP5 – pppGpp; Spot – origin).

Supplemental Figure 3

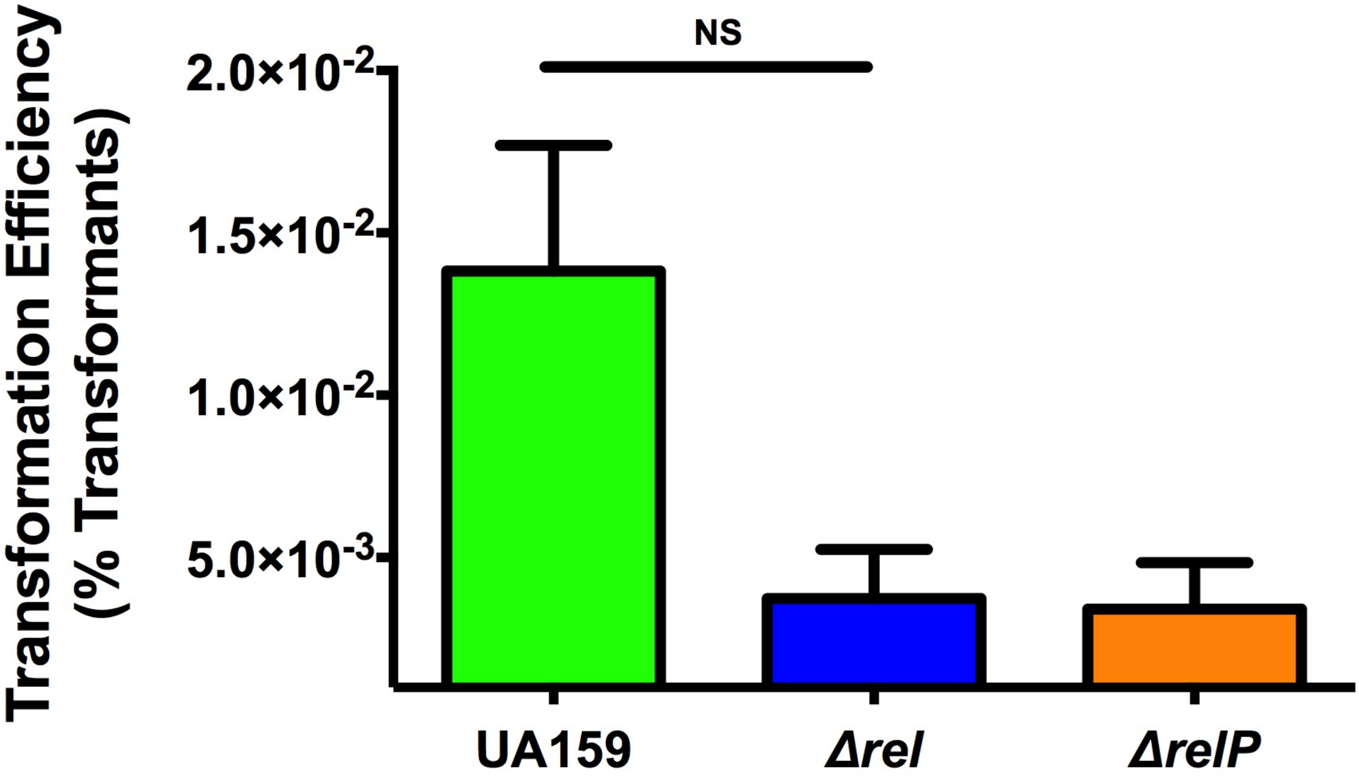

Fig. S3. Transformation efficiency in BHI after treatment with sCSP. Transformation efficiency of UA159, and the Δ*relA* or Δ*relP* mutants in the chemically complex medium BHI. After cultures reached an $OD_{600\ nm}$ = 0.2, 0.8 μM sCSP was added along with 500 ng of transforming DNA plasmid pDL278 (Sp^R). After 48 hours of incubation, CFUs were counted. Transformation efficiency was calculated by taking the number of transformants and dividing that by the total number of viable bacteria, then multiplying by 100 to obtain percent transformants in the population. Data are averages of three biological replicates with transformations performed in triplicate. Statistical analysis was performed by student's t-test. NS = not significant.

Supplemental Figure 4A – *comYA* mRNA

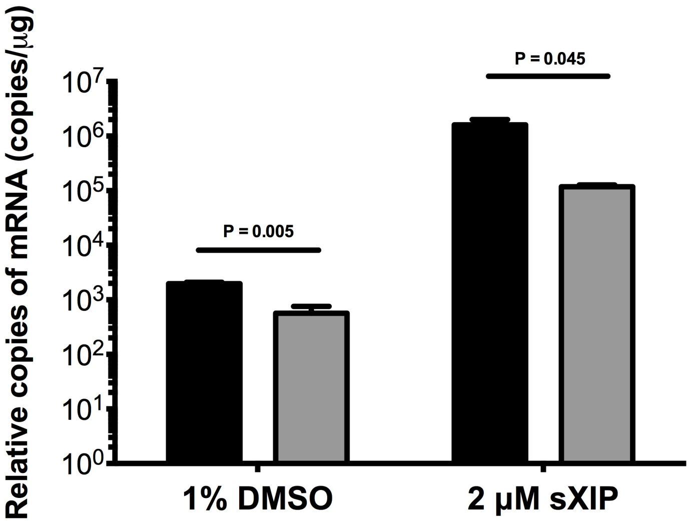

Supplemental Figure 4B – *comS* mRNA

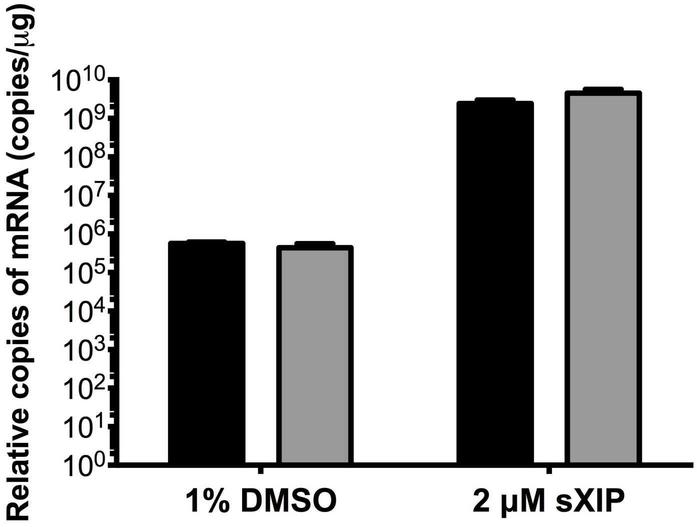

Supplemental Figure 4C – *comD* mRNA

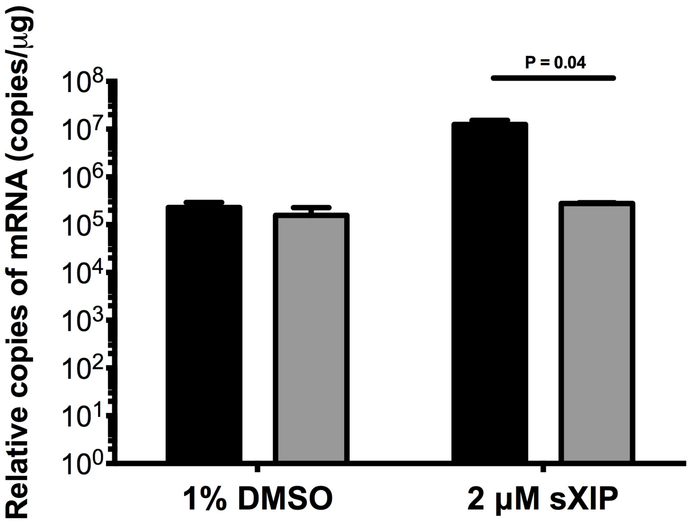

Supplemental Figure 4D – *cipB* mRNA

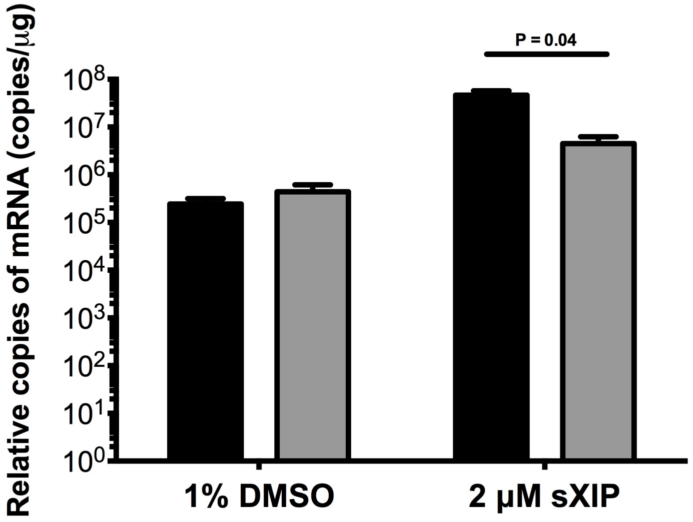

Fig. S4. Deletion of *relA* impacts *com* signaling. Differences in *com* gene expression between strains UA159 (black bars) and Δ*relA* (gray bars). After cultures reached an $OD_{600\ nm}$ = 0.2, 2 μM sXIP was added. After one hour of incubation, cells were harvested by centrifugation, RNA isolated, and qRT-PCR performed measuring (A) *comYA,* (B) *comS,* (C) *comD,* and (D) *cipB* mRNA. Gene expression was normalized to 16S rRNA expression. Data are averages of three biological replicates assayed in triplicate. Statistical analysis was performed by student's t-test.
